# C-Terminal Region of EBNA-2 Determines the Superior Transforming Ability of Type 1 Epstein-Barr Virus by Enhanced Gene Regulation of LMP-1 and CXCR7

**DOI:** 10.1371/journal.ppat.1002164

**Published:** 2011-07-28

**Authors:** Laila Cancian, Rachel Bosshard, Walter Lucchesi, Claudio Elgueta Karstegl, Paul J. Farrell

**Affiliations:** Section of Virology, Faculty of Medicine, Imperial College London, St. Mary's Campus, Norfolk Place, London, United Kingdom; Emory University, United States of America

## Abstract

Type 1 Epstein-Barr virus (EBV) strains immortalize B lymphocytes *in vitro* much more efficiently than type 2 EBV, a difference previously mapped to the EBNA-2 locus. Here we demonstrate that the greater transforming activity of type 1 EBV correlates with a stronger and more rapid induction of the viral oncogene LMP-1 and the cell gene CXCR7 (which are both required for proliferation of EBV-LCLs) during infection of primary B cells with recombinant viruses. Surprisingly, although the major sequence differences between type 1 and type 2 EBNA-2 lie in N-terminal parts of the protein, the superior ability of type 1 EBNA-2 to induce proliferation of EBV-infected lymphoblasts is mostly determined by the C-terminus of EBNA-2. Substitution of the C-terminus of type 1 EBNA-2 into the type 2 protein is sufficient to confer a type 1 growth phenotype and type 1 expression levels of LMP-1 and CXCR7 in an EREB2.5 cell growth assay. Within this region, the RG, CR7 and TAD domains are the minimum type 1 sequences required. Sequencing the C-terminus of EBNA-2 from additional EBV isolates showed high sequence identity within type 1 isolates or within type 2 isolates, indicating that the functional differences mapped are typical of EBV type sequences. The results indicate that the C-terminus of EBNA-2 accounts for the greater ability of type 1 EBV to promote B cell proliferation, through mechanisms that include higher induction of genes (LMP-1 and CXCR7) required for proliferation and survival of EBV-LCLs.

## Author Summary

Epstein-Barr virus (EBV) is a common human virus that is involved in several types of cancer and directly causes human B lymphocytes to proliferate when they become infected. EBV occurs naturally as two different viral types (type 1 and type 2). The genomes of these viruses are mostly very similar but they differ in a few genes, particularly the EBNA-2 gene. For many years it has been known that type 1 EBV is much more effective than type 2 EBV at causing B lymphocyte proliferation and this difference is mediated by the EBNA-2 gene. Here we have shown that the greater ability of type 1 EBNA-2 to cause B cell proliferation is due to superior induction of the EBV LMP-1 and the cell CXCR7 genes, both of which are required for growth of EBV-infected lymphocytes. We mapped the section of type 1 EBNA-2 responsible for this to the C-terminus of the protein, including the transactivation and EBNA-LP interaction domains. The results provide a mechanism for the long-standing question of the functional difference between these two major types of EBV and will be important in understanding the significance of the EBV types in human infection.

## Introduction

Epstein-Barr Virus (EBV) is a B-lymphotropic gamma herpesvirus which persistently infects over 90% of the adult population world-wide. EBV infection is usually asymptomatic, although in some cases the virus can be the causative agent of infectious mononucleosis [1]. EBV is also involved in some B cell cancers, such as Burkitt's Lymphoma (BL), Hodgkin's Lymphoma and lymphoproliferative disease in immunocompromised hosts, in addition to various epithelial tumors, for example nasopharyngeal carcinoma (NPC) and gastric cancer [2]. *In vitro*, EBV can efficiently immortalize primary B cells, converting them into permanently growing lymphoblastoid cell lines (LCLs), in which the viral genome is maintained in an episomal form. A specific viral gene expression program, known as latency III [3], is activated in LCLs and this involves expression of six nuclear antigens (EBNA-1, -2, -3A, -3B, -3C and -LP), three membrane proteins (LMP-1, -2A and -2B), and various non-coding small RNAs (EBERs and miRNAs). Together these viral gene products activate the resting B cells and sustain their continuous proliferation. [1]. Work with recombinant EBV viruses has demonstrated that at least five of the latent proteins (EBNA-1, -2, -3A, -3C and LMP-1) are essential for EBV-mediated B cell transformation, while EBNA-LP and LMP-2 can contribute to optimal efficiency of this process [4]-[11].

EBV isolates can be classified as type 1 or type 2 (also known as type A and B) based on linked sequence variation in the latent genes EBNA-2, EBNA-3A, -3B, -3C and EBNA-LP. The most divergent locus between the two viral types is EBNA-2, which defines the viral types. There is about 50% difference in the predicted primary amino acid sequence, which is strikingly high if compared to the extremely low degree of variation in the rest of the EBV genome [12]–[17]. Type 1 strains of EBV are ubiquitous in the world, whereas type 2 strains are frequent in some areas of Central Africa and some other parts of the world where malaria is endemic. In these regions, type 1 and type 2 EBV have approximately equal prevalence [18]. The major biological difference between the two viral types is that type 1 EBV immortalizes B cells *in vitro* much more efficiently than type 2 EBV [19]. Experiments with a recombinant type 2 EBV virus carrying a type 1 EBNA-2 sequence showed that this virus gained a type 1 immortalization phenotype, demonstrating that the difference in transformation efficiency is determined by the EBNA-2 locus [5]. The *in vitro* transforming activities of type 1 and type 2 EBV also correlate with the frequency of tumor formation in SCID mice inoculated with type 1 or type 2 EBV *in vitro*-transformed LCLs [20], [21]. Despite the clear difference in growth phenotype *in vitro*, there is so far no obvious disease association or *in vivo* phenotype known for type 1 and type 2 EBV strains, although one study reported that type 1 EBV strains are significantly more likely to cause infectious mononucleosis, compared to type 2 strains [22].

Upon EBV infection of *naïve* B cells *in vitro*, EBNA-2 and EBNA-LP are the first viral latent proteins expressed. EBNA-2 is a transcription factor that can activate expression of the viral Cp and LMP promoters and many cell genes crucial for B cell survival and proliferation [1], [23]–[26]. Activation of some of these promoters by EBNA-2 is enhanced by cooperation with EBNA-LP [27]-[31]. Sequence comparison analysis of the EBNA-2 allele from type 1 and type 2 EBV and different primate lymphocryptoviruses, led to identification of nine evolutionary conserved regions (CR1 to CR9) which represent much of the total sequence homology between type 1 and type 2 EBNA-2 proteins and define some functional domains and important structures of the protein [13], [32] (see diagram below). CRs 1 to 4 at the N-terminus of the protein correspond to two self-association domains [33], [34] and a poly-proline region, which consists of a variable number of consecutive proline residues, depending on the virus strain. CRs 5 to 9 are located in the C-terminal half of the EBNA-2 protein, separated from the N-terminal cluster of CRs by the diversity region, where the sequence similarity between type 1 and type 2 EBNA-2 is very low. CR5 is involved in association with the host DNA-binding protein RBP-Jk and CR6 mediates the interaction with the cell protein SKIP, which facilitates EBNA-2/RBP-Jk complex formation [35], [36]. CR8 is part of an acidic transactivation domain (TAD), which mediates gene transcriptional activation, whereas CR9 coincides with a nuclear localization signal (NLS). An additional karyophilic signal is represented by the RG sequence, which consists of an 18-amino acid stretch rich in arginines and glycines [32]. Four domains of EBNA-2 have been shown to be involved in EBNA-2/EBNA-LP cooperation: the TAD, amino acids 1 to 58, the RG motif and the CR7 [31], [37]. Detailed mutational analysis of the type 1 EBNA-2 protein led to mapping the regions essential for transformation. These regions are mostly also essential for transactivation of the LMP-1 promoter, suggesting that transformation and transactivation functions of EBNA-2 are closely related [38]. In the N-terminal half of EBNA-2, residues 3 to 30 have been shown to be required for induction of LMP-1 expression and, consequently, for immortalization maintenance, using the EREB2.5 *trans*-complementation system [39]. CR4 has been shown to contribute to EBNA-2 mediated immortalization of B cells, as mutant viruses with a deleted CR4 are drastically impaired in B cell transformation [40]. B cell infection experiments with recombinant viruses suggested that a minimum of seven prolines in the poly-proline region is required for transformation, whereas in the EREB2.5 *trans*-complementation assay, the whole poly-proline region was found to be dispensable for EBNA-2-mediated immortalization maintenance [41], [42]. In the C-terminal half of the protein, the transactivation domain and the RBP-Jk-binding region are absolutely required for B cell immortalization and LMP-1 induction [38], [43]. The RG motif has been demonstrated to be important for optimal B lymphocyte transformation efficiency [44]. However, a deletion mutant EBNA-2 protein lacking the RG sequence displayed a four-fold increase in the activation of the LMP-1 promoter in reporter assays, compared to wild-type EBNA-2, suggesting that the RG domain is a negative regulator of EBNA-2 activity on the LMP-1 promoter [44].

EBNA-2 mechanisms of transcriptional activation have been identified by many studies, which have mainly involved type 1 EBNA-2, whereas less is known about the type 2 EBNA-2. EBNA-2 does not bind directly to DNA but is tethered to EBNA-2 responsive promoters by interacting with various cell DNA-bound transcription factors. For example, it interacts through its conserved WWPP motif with the transcriptional repressor RBP-Jk (CBF1), thereby converting RBP-Jk to the transcriptionally active form [35], [45]. Additional cell sequence-specific transcription factors are involved in EBNA-2 recruitment at some target promoters, such as Spi-1/PU.1, AP-2 and AUF1 [46]–[50]. EBNA-2-mediated gene transcription is activated by the transactivation domain which interacts with several cell basal transcription factors (TFIIH, TAF40, TFIIB and p100/TFIIE) and histone acetyltransferases (p300/CBP and PCAF) [51]–[54]. Other EBNA-2-interacting partners are involved in chromatin remodelling, such as proteins of the cell SWI-SNF complex [55], [56].

EBNA-LP contains multiple copies of an N-terminal 66-aa domain encoded by two exons (W1 and W2), located within each of the BamHI W repeats of the EBV genome, and a unique C-terminal 45-aa domain encoded by the Y1 and Y2 exons within the downstream BamHI Y fragment [57]–[59]. Due to variations in the number of BamHI W repeats in different viruses and alternative splicing between the repeated W1W2 exons and the unique Y1Y2 exons, EBNA-LP proteins of different sizes can be expressed in EBV-infected cells. Genetic studies showed that recombinant viruses lacking the C-terminal 45 amino acids display a markedly lower immortalization efficiency compared to wild-type viruses and require feeder cells, suggesting that EBNA-LP is important but not essential for EBV-induced immortalization [6], [9]. Importantly, EBNA-LP has been shown to cooperate with EBNA-2 and enhance EBNA-2 transcriptional activation of both viral (LMP-1/LMP-2B, Cp) and cell (cyclin D2) target promoters [27]–[31]. Most of the cooperative function of EBNA-LP maps to the W1W2 repeats, whereas the unique carboxyl-terminal Y1Y2 domains modulate EBNA-LP function [28], [29]. Only proteins with 2 or more copies of the W1W2 repeat are capable of cooperating with EBNA-2 and viruses with two BamHI W repeats are immortalization-competent [17], [29], [60], [61].

LMP-1 is required for establishment of B cell transformation *in vitro* [8] and is also required for continuous proliferation of EBV-infected LCLs [62]. Regulation of LMP-1 by EBNA-2 is complex and involves many cell proteins, including RBP-Jk, PU.1, AP-2, SWI-SNF, CBP/p300, ATF/CREB [46]-[49], [54], [63]. Unlike other EBNA-2 target promoters (e.g. LMP-2A), the EBNA-2/RBP-Jk interaction plays only a minor role in EBNA-2-induced activation of the LMP-1 promoter [64]. Since the EBNA-2 domains that are essential for B cell transformation and LMP-1 induction are similar, transactivation of LMP-1 by EBNA-2 is considered to play a key role in EBNA-2-induced B lymphocyte transformation [65].

Several studies have used microarray analysis to identify human genes that are targets of type 1 EBNA-2 [23]–[25] but until recently little was known about the ability of type 2 EBNA-2 to regulate gene expression. In earlier reports the abilities of type 1 and type 2 EBNA-2 to up-regulate gene expression were compared only on two individual promoters, LMP-1 and CD23 [66], [67]. Recently we compared the host genes induced by type 1 EBNA-2 to those induced by the type 2. Only a few genes were found to be differentially regulated (CXCR7, MARCKS, IL1β and ADAMDEC), with a stronger induction by type 1 EBNA-2 [26]. Among these, CXCR7 was the most differentially regulated gene and was also shown to be required for proliferation of EBV-infected LCLs. Expression of MARCKS, IL1β and ADAMDEC was very low in LCLs and may not be significant [26]. CXCR7 (RDC1, CMKOR1) is a G-protein coupled chemokine receptor with seven trans-membrane domains [68], [69]. CXCR7 binds with high affinity to the inflammatory and homing chemokines CXCL12/SDF-1 and CXCL11/ITAC, which are also ligands for CXCR4 and CXCR3 respectively [70], [71]. CXCR7 is believed to be an atypical chemokine receptor since neither coupling of the receptor to G-proteins nor CXCR7-mediated triggering of typical chemokine responses, such as chemotaxis or receptor-induced calcium mobilization, could be demonstrated [70]-[73]. Therefore, CXCR7 has been proposed to be mainly a modulator of CXCR4 and CXCR3 signalling, possibly acting as a “decoy” receptor to scavenge and sequester CXCL12/SDF-1 and CXCL11/ITAC [74], [75]. This is suggested by the observation that CXCR7 expression on mature B cells inversely correlates with the activity of CXCR4 [76]. Alternatively, a function for CXCR7 as co-receptor for CXCR4 has also been proposed, as the two receptors can heterodimerize [77]. Analysis of CXCR7 expression in normal human leukocytes revealed that the mRNA is broadly expressed in various fractions of resting PBMCs, including B cells [76], [78], whereas the protein seems to be strictly expressed at the plasma membrane of monocytes and B cells [76]. However, surface CXCR7 was not consistently detected on B cells in another study [78]. CXCR7 has been implicated in tumorigenesis based on the observation that ectopic expression of the gene induces tumor formation in nude mice [79]. Moreover, CXCR7 protein has been shown to be expressed on many human and mouse tumor cell lines and to confer a strong growth and survival advantage to the cells [71], [80]–[82].

In this study we show that LMP-1 and CXCR7 mRNAs are differentially induced by type 1 and type 2 EBV during the early stages of infection of primary B cells with recombinant EBV viruses, consistent with differential induction of these genes being the basis for the weaker ability of type 2 EBV strains to transform primary B cells. EBNA-2 type, rather than the type of EBNA-LP, is shown to be the major determinant of the differential induction of LMP-1 oncoprotein and the C-terminal region of EBNA-2 is responsible for the superior ability of the type 1 protein to maintain proliferation of B cells. Within this region, the RG, CR7 and TAD are the minimum type 1 domains required for type 1 growth phenotype and sufficient LMP-1/CXCR7 expression levels. The results demonstrate a mechanism for the enhanced capability of type 1 strains of EBV to transform B cells into LCLs.

## Results

### Differential regulation of LMP-1 and CXCR7 mRNA by ER-EBNA-2 shows that type 1 EBNA-2 induces LMP-1 and CXCR7 to a higher level and more rapidly than type 2 EBNA-2

**Figure 1:**
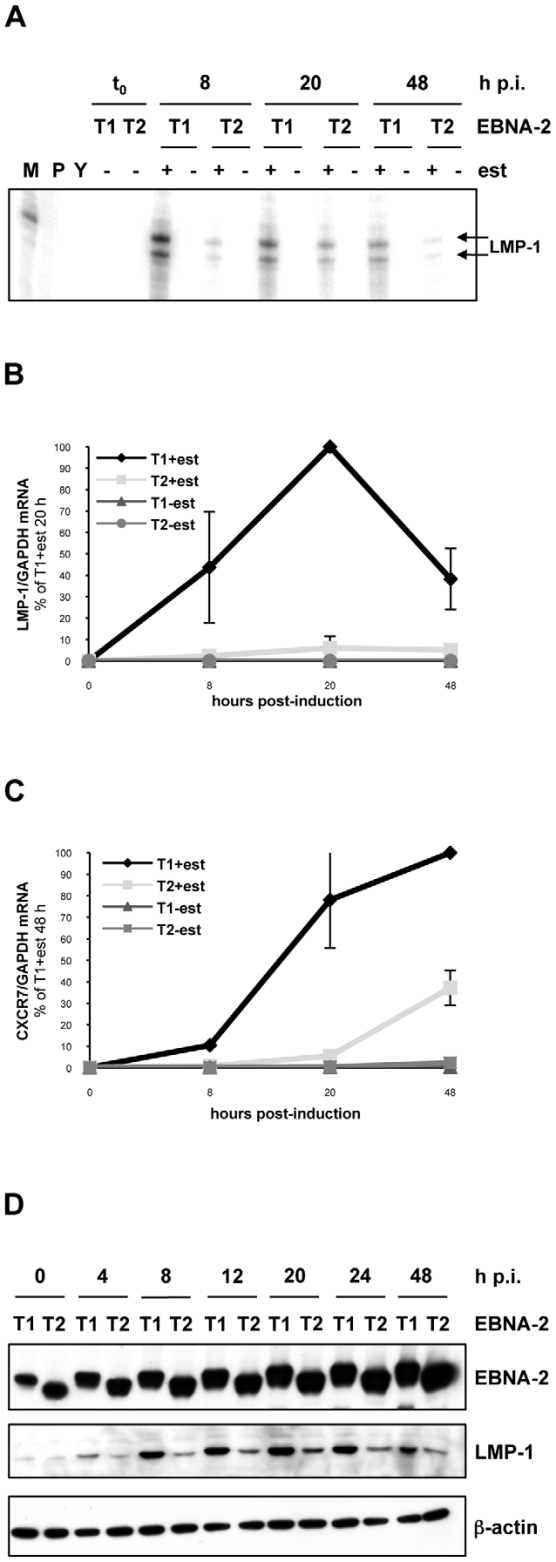
LMP-1 and CXCR7 are differentially induced by type 1 and type 2 EBNA-2 in Daudi:ER-EBNA-2 T1/T2 and P3HR1 cl.16:ER-EBNA-2 T1/T2 stable cell lines. (**A**) (**B**) (**C**) 20×10^6^ Daudi cells stably expressing type 1 (T1) or type 2 (T2) ER-EBNA-2 were induced with 1 µM oestrogen (+) or ethanol (-) as a solvent control, for 8, 20 or 48 hours. (**A**) RPA for LMP-1 mRNA. 20 µg of total cell RNA were used. The doublet protected fragment of around 175 nt, is marked by arrows. M: size marker, the 179 nt band is shown; P: undigested probe; Y: yeast RNA (RPAIIIkit, Ambion). 1 representative experiment of 3 is shown. (**B**) **and** (**C**) LMP-1 and CXCR7 mRNA qRT-PCR analysis. 1 µg of total cell RNA was used to synthesize cDNA and qPCR was performed to quantify LMP-1 and CXCR7 transcripts. For LMP-1 qRT-PCR, values for each sample are expressed as the ratio LMP-1/GAPDH mRNA relative to the sample T1 stimulated with oestrogen for 20 hours, whereas for CXCR7 qRT-PCR values shown represent the ratio between CXCR7 and GAPDH mRNA levels, relative to the sample T1 stimulated with oestrogen for 48 hours. Each time-point corresponds to the average from 2 independent experiments both performed in duplicate. Error bars represent standard errors. Data shown in panels (**B**) **and** (**C**) and results presented in panel (**A**) are from different experiments. (**D**) P3HR1 cl.16 cells stably expressing type 1 (T1) or type 2 (T2) ER-EBNA-2 were induced with 1 µM oestrogen (+) or ethanol (-) as a solvent control, for 4, 8, 12, 20, 24 or 48 hours. At these time-points proteins were extracted and analyzed by western blotting for EBNA-2 (PE2 antibody), LMP-1 (CS 1-4 antibody) and β-actin, as a loading control. h p.i.: hours post-induction; est: oestrogen.

We previously identified CXCR7 as the most differentially regulated type-specific EBNA-2-target gene in the human genome, being more strongly induced by type 1 EBNA-2 in a BL cell background [26]. RNAi experiments demonstrated that CXCR7 is required for proliferation of EBV-LCLs [26]. We also showed, in an EBV-positive BL cell line with oestrogen-regulated EBNA-2 proteins, that type 1 EBNA-2 induced LMP-1 protein more rapidly and to a higher extent compared to the type 2 [26]. This has been now verified at the RNA level using the same system. Briefly, this consists of Daudi cells (which lack the EBNA-2 locus [83] and therefore expression of LMP-1) engineered to stably express type 1 or type 2 chimaeric EBNA-2 proteins fused to the hormone-binding domain of the oestrogen receptor (ER-EBNA-2) [26]. In the absence of oestrogen, the ER-tagged EBNA-2 proteins are localized to the cytoplasm and therefore inactive [84]. Addition of oestrogen results in normal localization of the EBNA-2 fusion proteins to the nucleus and therefore in activation of the LMP-1 gene from the resident EBV genome. In a time-course experiment of oestrogen stimulation over 48 hours, total cell RNA from the cell lines containing type 1 or type 2 ER-EBNA-2 was analyzed by ribonuclease protection assay (RPA) using an LMP-1 specific probe (Figure 1 A). When type 1 EBNA-2 function was activated, LMP-1 mRNA levels were strongly induced as soon as 8 hours after the stimulation and were maintained thereafter throughout the time-course. In contrast, after induction of type 2 EBNA-2 function, markedly lower levels of LMP-1 transcripts were detected. Analysis by qRT-PCR for LMP-1 mRNA gave a similar result (Figure 1 B) and qRT-PCR analysis was also performed for CXCR7 transcripts levels in the same experiment (Figure 1 C). These were found to be significantly higher in type 1 EBNA-2 cell lines stimulated with oestrogen than in type 2 cells that had undergone the same treatment. Agarose-gel electrophoresis analysis of total cell RNA used for the RPA analysis confirmed equal loading and no degradation of the samples (data not shown). The somewhat higher expression of type 2 ER-EBNA-2 in the Daudi cell line (Figure S1 and [26]) further emphasizes the superior induction of target genes by type 1 ER-EBNA-2.

The more rapid induction of LMP-1 by type 1 EBNA-2 was also confirmed in the type 2 EBV P3HR1 cl.16 BL cell line. The resident EBV genome in P3HR1 cl.16 line also lacks the EBNA-2 locus and the Y1Y2-coding region of EBNA-LP, having a deletion similar but not identical to that present in Daudi cells [83], [85]. Plasmids expressing type 1 or type 2 ER-EBNA-2 (p554-4 and its type 2 derivative, described in Materials and Methods and in [26], [86]) were transfected into P3HR1 cl.16 cells and stable cell lines were selected with G418. In P3HR1 cl.16 cells, despite the absence of EBNA-2, basal expression of LMP-1 can still be detected, albeit at very low levels (Figure 1 D). Nevertheless, when a time-course experiment of oestrogen stimulation was performed, a clear increase of LMP-1 expression levels above background was detected by western blotting when type 1 EBNA-2 function was activated (Figure 1 D). LMP-1 induction was detected as soon as 4 hours after oestrogen stimulation; after 8 hours LMP-1 expression level reached its maximum and was thereafter maintained throughout the time-course. Conversely, when type 2 EBNA-2 function was activated by oestrogen, LMP-1 levels were induced only moderately above the background, with a modest peak at 20 hours followed by a slight decrease (Figure 1 D).

### Weaker induction of LMP-1 by type 2 EBNA-2 is not affected by the EBNA-LP type

As EBNA-LP is important in cooperation with EBNA-2 for induction of LMP-1 [28], [29], we determined which isoforms of EBNA-LP are expressed in normal Daudi and P3HR1 cl.16 cells and in the derivative stable cell lines bearing the oestrogen-inducible EBNA-2 proteins (Figures S1 and S2). In several previous reports, depending on the antibodies used, one or two species of EBNA-LP (around 31 and 37 kDa) have been detected in Daudi cells, both lacking the Y1Y2 domain [87]–[90]. Here we used the 4D3 antibody and observed the 37 kDa isoform (Figure S1, lane 4), which corresponds to a 4-repeat species, as determined by comparison with the EBNA-LPs expressed in B95-8 and in 293 cells transiently transfected with plasmids coding for 3- or 7- repeat EBNA-LP as standards (Figure S1, lanes 1, 2 and 3). P3HR1 cl.16 cells have been reported to express Y1Y2-deleted EBNA-LP species migrating as a major band of around 28–30 kDa and a minor band of about 48–50 kDa on a SDS-PAGE [91] and this was confirmed by our western blot analysis with the 4D3 antibody (Figure S2, lane 4). We identified these species as containing 3 and 6 W repeats, respectively (by comparison with B95-8). In both cell lines these truncated EBNA-LP proteins are type 2, since they were detected with the 4D3 antibody, which detects both type 1 and type 2 EBNA-LP [89], but not with the JF186 antibody, which is type 1-specific [92]. In the stable cell lines expressing ER-EBNA-2 proteins several EBNA-LP isoforms were detected, with 2 to 4 repeats in Daudi:ER-EBNA-2 T1/T2 (Figure S1, lanes 5 to 8) and with 2 to 7 repeats in P3HR1 cl.16:ER-EBNA-2 T1/T2 (Figure S2, lanes 5 to 8). These species are type 1, since they can be detected with the JF186 antibody, and are expressed from the plasmids that code for the ER-EBNA-2 proteins (p554-4 and its type 2 derivative [26], [86]). In fact, these vectors contain not only the ER-EBNA-2 open reading frame, but also 2 W repeats and the Y fragment, both of type 1 sequence. The expression of EBNA-LP species that are larger in size than that predicted simply from the number of W fragments has been already reported by others [92]-[94] and may indicate splicing of RNA transcribed more than once around the plasmid. In both systems (Daudi and P3HR1 cl.16:ER-EBNA-2 T1/T2) several isoforms of full-length type 1 EBNA-LP are present but no full-length type 2 EBNA-LP is available.

**Figure 2:**
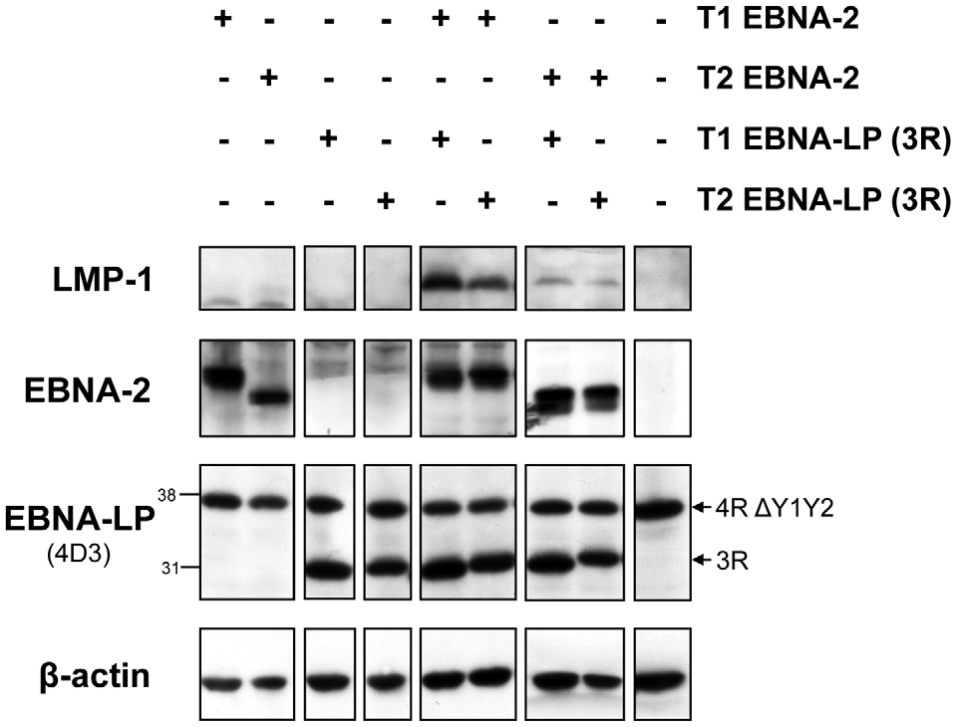
In a transient transfection assay in Daudi cells, the weaker induction of LMP-1 by type 2 EBNA-2, compared to the type 1 EBNA-2, is not affected by the EBNA-LP type. 2×10^6^ Daudi cells were transiently transfected with an array of combinations of EBNA-2-expressing plasmids (OriP-p294) and EBNA-LP-expressing plasmids (pSNOC) of type 1 (T1) and type 2 (T2). The Neon transfection system (Invitrogen) was used. 24 hours after transfection, cells were lysed in RIPA lysis buffer and analyzed by SDS-PAGE followed by immunoblotting for LMP-1 (CS 1-4 antibody), EBNA-2 (PE2 antibody), EBNA-LP (4D3 antibody) and β-actin (to ensure equal loading of the proteins). The pSNOC plasmids express EBNA-LP proteins with 3 W repeats (3R). Daudi cells already express an endogenous species of EBNA-LP which bears 4 repeats and lacks the Y1Y2 domains (4R ΔY1Y2). Numbers next to the EBNA-LP blot indicate protein molecular weight in kDa. 1 representative experiment of 2 is shown.

To exclude the possibility that type 1 EBNA-LP proteins are able to enhance the ability of type 1 ER-EBNA-2 to induce LMP-1 but not that of the type 2 ER-EBNA-2, we performed an assay for EBNA-LP function, similar to that used in earlier studies [17], [29], whereby the effect of EBNA-LP on EBNA-2-induced expression of LMP-1 from the resident EBV genome can be examined. This assay was adapted to compare the cooperative effect of type 1 and type 2 EBNA-LP on type 1 and type 2 EBNA-2-induced expression of LMP-1 in Daudi cells. Transfection of vectors expressing type 1 or type 2 EBNA-2 alone did not produce any significant induction of LMP-1, despite the strong expression of both types of EBNA-2 proteins, as assessed by immunoblotting (Figure 2). Likewise, no LMP-1 was detected when plasmids coding for either type 1 or type 2 EBNA-LP bearing 3 repeats were transfected, even though both EBNA-LP species were clearly detected on a western blot as an approximately 30 kDa band. Co-transfection of type 1 EBNA-2 and type 1 EBNA-LP plasmids produced a clear induction of LMP-1 protein. A similar, but slightly lower level of induction was observed when the type 2 EBNA-LP was co-expressed with type 1 EBNA-2. In contrast, up-regulation of LMP-1 induced by the type 2 EBNA-2, in the presence of either type of EBNA-LP, was very modest and equal with either type 1 or type 2 EBNA-LP (Figure 2). These results indicate that the weaker LMP-1 up-regulation induced by type 2 EBNA-2 can be ascribed to the EBNA-2 type and is not affected by the type of EBNA-LP present. Type 1 EBNA-2 up-regulates LMP-1 better than type 2 EBNA-2, in the presence of either type 1 or type 2 EBNA-LP. These observations support the conclusion that, in the Daudi and P3HR1 cl.16:ER-EBNA-2 T1/T2 cell lines, the impaired ability of type 2 EBNA-2 to induce LMP-1 is not due to sub-optimal cooperation with the full-length type 1 EBNA-LP isoforms present in the systems, but is rather due to the type 2 EBNA-2 protein.

### Differential regulation of LMP-1 and CXCR7 genes during early stages of infection of primary B cells with BAC-derived EBV expressing type 1 EBNA-2 or type 2 EBNA-2

**Figure 3:**
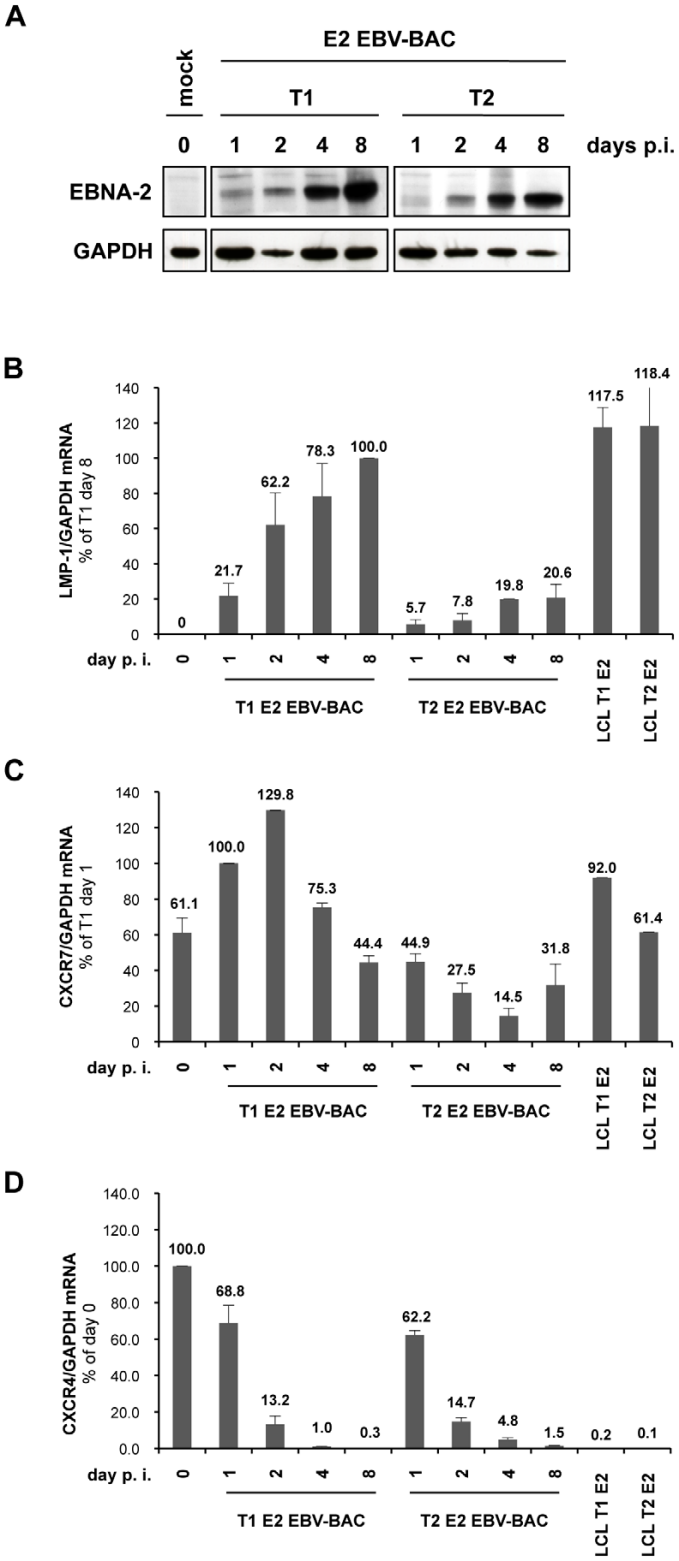
Differential regulation of LMP-1 and CXCR7 genes during early stages of infection of primary B cells with BAC-derived EBV expressing type 1 EBNA-2 or type 2 EBNA-2. Primary B cells were isolated from peripheral blood by negative selection using RosetteSep (Stemcell Technologies). 2×10^6^ cells were infected with 5000 GRUs of either T1 E2 EBV-BAC (expressing type 1 EBNA-2) or T2 E2 EBV-BAC (expressing type 2 EBNA-2) recombinant viruses. Cells were harvested at 1, 2, 4 and 8 days post-infection and processed for protein and total RNA extraction. Uninfected cells, harvested at day 0, were included in the analysis. (**A**) Whole protein extracts from each well of infected cells were loaded on an SDS-PAGE gel and subjected to western blot analysis using the anti-EBNA-2 antibody (PE2 clone). The blots were re-probed for GAPDH to show the total amount of protein recovered from each sample. GAPDH levels at 4 and 8 days post-infection were lower in type 2-infected cells compared to type 1-infected cells, due to faster proliferation of the latter. (**B**) (**C**) **and** (**D**) qRT-PCR analysis of total cell RNA samples using LMP-1, CXCR7 and CXCR4 specific primers. RNAs from LCLs established with T1 and T2 EBNA-2 EBV-BAC were included in the analysis (LCL T1 E2 and LCL T2 E2). For LMP-1 qRT-PCR (**B**), values for each sample are expressed as the ratio LMP-1/GAPDH mRNA relative to the sample T1 day 8; for CXCR7 qRT-PCR (**C**), values shown represent the ratio between CXCR7 and GAPDH mRNA levels relative to the sample T1 day 1; for CXCR4 qRT-PCR (**D**), values are reported as the ratio CXCR4/GAPDH mRNA relative to mock infected cells. The qRT-PCR experiment was performed in duplicate and error bars represent standard errors. 1 representative experiment of 4 is shown. p.i.: post-infection.

Having showed, in our previous report [26] and in the present study in assays in cell lines, that type 1 and type 2 EBNA-2 differentially regulate LMP-1 and CXCR7, we examined whether this phenomenon occurs also during infection of primary B cells with BAC-derived EBV recombinant viruses, expressing either type 1 or type 2 EBNA-2. Early stages of infection were studied since the differences in kinetics of gene regulation induced by the two types of EBNA-2 appeared rapidly in our cell line-based systems. Normalized amounts of the EB viruses expressing type 1 or type 2 EBNA-2 (described in Materials and Methods) were used to infect aliquots of 2×10^6^ human primary B cells, purified from peripheral blood. Samples were taken at 1, 2, 4 and 8 days post-infection and examined by western blotting and quantitative RT-PCR (qRT-PCR) for EBNA-2-target gene expression. Similar amounts of type 1 and type 2 EBNA-2 proteins were expressed, as determined by the EBNA-2 immunoblot (Figure 3 A). In type 1 EBV-infected cells, LMP-1 transcripts were detected as soon as 2 days post-infection and gradually increased thereafter reaching typical LCL levels of expression by day 8, whereas in type 2-infected cells LMP-1 induction was delayed, with overall lower levels than those observed in type 1 infection (Figure 3 B). These different kinetics of LMP-1 mRNA induction were consistently observed in 4 independent infection experiments, although the overall level of expression and precise timing varied slightly in different experiments, this variability perhaps being due to different B cell donors being used each time. As we were able to perform these experiments only on a small scale, LMP-1 protein could not be detected by western blotting at these early times after infection.

qRT-PCR analysis for CXCR7 mRNA revealed that uninfected resting B cells (harvested at day 0) already expressed substantial basal amounts of mRNA (Figure 3 C), an observation consistent with other reports [76], [78]. Following viral infection, CXCR7 mRNA increased up to 2 days after infection and then settled back to the LCL level (Figure 3 C). On the other hand, in type 2 infection the mRNA for CXCR7 was consistently at about a 4-fold lower level than in type 1 virus-infected cells (Figure 3 C). This trend in CXCR7 mRNA levels was observed in 3 independent experiments, with minor differences in the overall levels of expression.

Expression of the chemokine receptor CXCR4, which shares the ligand CXCL12/SDF-1 with CXCR7, has been shown to be down-regulated in primary B cells upon infection by EBV [95]-[97]. Uninfected primary B cells were found to express high levels of CXCR4 mRNA (Figure 3 D), in agreement with other reports [98]. Upon viral infection, CXCR4 transcript levels were effectively repressed in cells infected with both type 1 and type 2 EBNA-2 recombinant EB viruses (Figure 3 D).

In order to establish LCLs, serial dilutions of preparations of EBV-BACs expressing type 1 or type 2 EBNA-2 were used to infect primary B lymphocytes (10^6^ cells) and their outgrowth was monitored over time. As expected, type 1 EBV-BAC-infected cells grew very rapidly, leading to LCLs within approximately 1 month post-infection, whereas type 2 transformants were much more difficult to expand and yielded cell lines only after approximately 4 months of culture. The growth phenotype observed with our recombinant type 1 and type 2 EBNA-2-expressing viruses is consistent with the growth behaviour originally reported for the natural strains of EBV B95-8 (type 1 prototype) and AG876 (type 2 prototype) [19]. These differences in growth kinetics, which were revealed by microscopic observation of the cells in culture, were mirrored by the counts of proliferating cells assessed at a single time-point after infection, for each of the cell lines originally infected with serial dilutions of virus (Figure S3). For example, the amount of live cells counted in type 1 LCLs originally infected with either 1600 or 320 GRUs of viral preparation was increased compared to the corresponding type 2 transformants by 53 and 22-fold, respectively (Figure S3). Once LCLs were established, hygromycin selection was applied to ensure the cells contained the BAC-EBV constructs, which bear the hygromycin-resistance gene, and not an EBV genome that might potentially have been endogenous at a low level in the donor B cells. The fully established type 1 and type 2 EBNA-2 LCLs were then further checked by examining the pattern of expression of all the EBV latent antigens, using western blotting (Figure S4). The EBV latent antigens examined are expressed at similar levels and display the expected size. EBNA-LP isoforms with slightly variable number of W repeats (from 3 to 5) were detected in type 1 and type 2 LCLs, but this is well above the minimum required for cooperation with EBNA-2 [17], [60]. Some differences were observed in EBNA-3A and -3C expression levels among the several clones analyzed, but do not seem to be consistently associated with one specific type of LCL (Figure S4).

### Mapping the regions of type 1 EBNA-2 which confer higher B cell immortalization efficiency compared to type 2 EBNA-2

**Figure 4:**
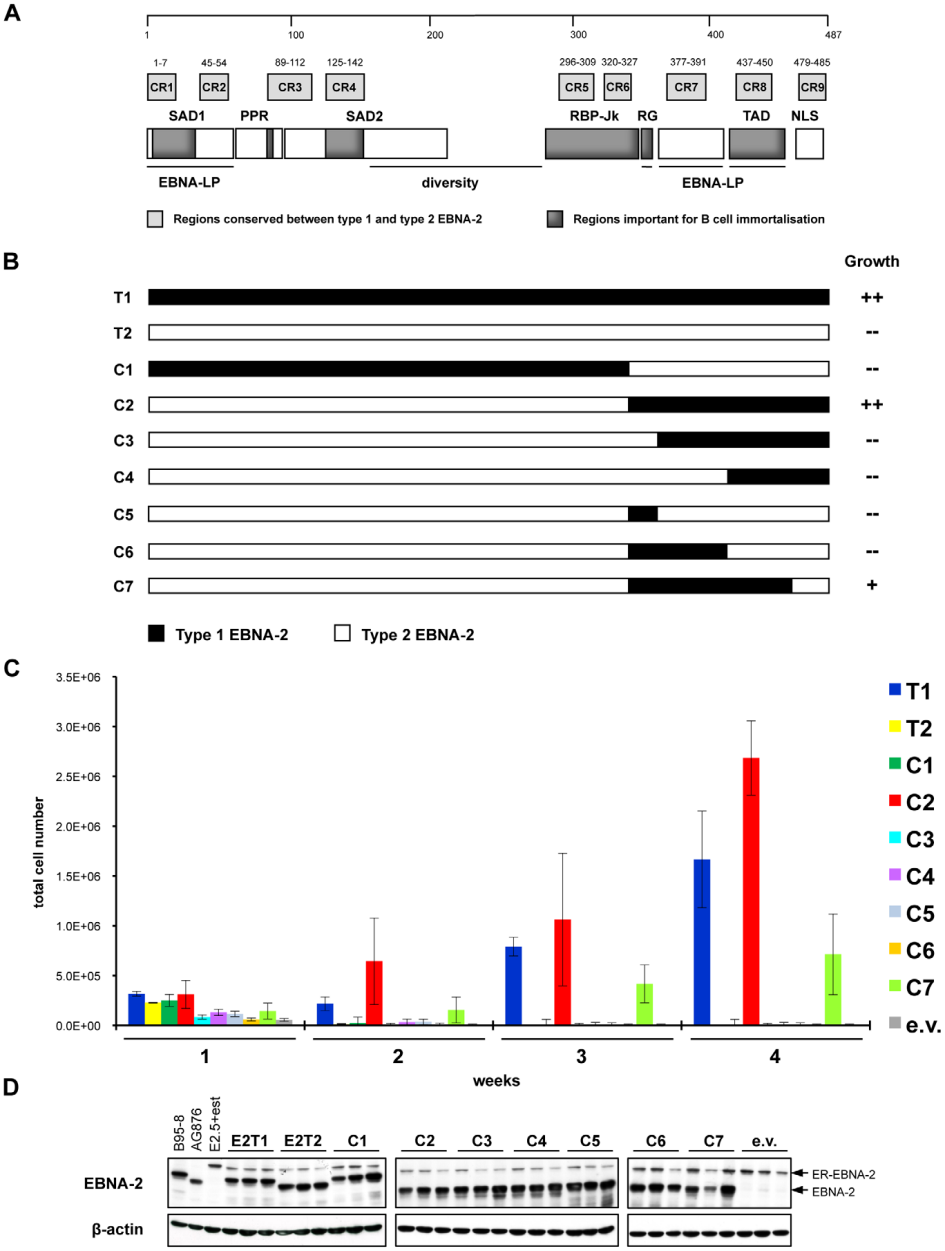
Type 1/type 2 EBNA-2 chimaeras tested in the EREB2.5 growth assay. The carboxyl-terminal region of type 1 EBNA-2 complements the deficiency of type 2 EBNA-2 in the EREB2.5 growth assay. RG, CR7 and TAD domains are the minimum type 1 sequences required. (**A**) Structure of EBNA-2 protein. Type 1 EBNA-2 protein of the prototype B95-8 strain is 487 amino acids long, whereas the type 2 protein from the strain AG876 comprises 455 amino acids. The two types of EBNA-2 proteins share around 50% of sequence identity, which consists mainly of 9 short stretches of homology, named conserved regions (CR1 - CR9). Characteristic parts of the EBNA-2 protein are: two N-terminal self-association domains (SAD1 and SAD2); a poly-proline region (PPR); a diversity region, a sequence with low similarity between EBV types; a region interacting with the cell DNA-binding protein RBP-Jk (RBP-Jk); a short sequence rich in arginine and glycine residues (RG); a transactivation domain (TAD); a carboxyl-terminal nuclear localisation signal (NLS). EBNA-LP indicates regions involved in cooperation between EBNA-2 and EBNA-LP in transcriptional activation. (**B**) Panel of chimaeric proteins (C1 - C7) tested for their ability to rescue proliferation of oestrogen-starved EREB2.5 cells. The growth phenotype was scored as “++” (type 1 and chimaera 2) and “+” (chimaera 7), when cell proliferation was maintained over time after transfection and oestrogen withdrawal and LCLs were successfully established, or as “–” (type 2, chimaeras 1, 3, 4, 5 and 6), when proliferation ceased after transfection. (**C**) Live cell counts of EREB2.5 cells expressing wild-type type 1/type 2 or chimaeric EBNA-2 proteins. EREB2.5 cells were transfected with OriP-p294 plasmids expressing wild-type type 1 (T1), type 2 (T2) EBNA-2, chimaeras 1 to 7 (C1 - C7) or with empty vector (e.v.). Oestrogen was removed from the culture medium and accumulation of proliferating cells was assessed at 1, 2, 3 and 4 weeks after transfection by counting cells that exclude Trypan Blue on a haemocytometer. Data from 1 representative experiment of at least 4 is shown. Transfections were performed in duplicate and for each replica, triplicate wells of cells were counted. Error bars indicate standard deviations. (**D**) Western blot analysis of protein extracts from EREB2.5 cells transfected with wild-type type 1 (E2T1) and type 2 (E2T2) and chimaeric (C1 - C7) EBNA-2 proteins. Cells were harvested 4 days after transfection. EBNA-2 was detected using the PE2 antibody, which recognizes both types. All the chimaeric EBNA-2 proteins were expressed at levels comparable to wild-type type 1 and type 2 and displayed the expected size, which corresponds to 85 kDa (similar to type 1) for chimaera 1 and to 75 kDa (similar to type 2) for all the other chimaeras. ER-EBNA-2 fusion protein (120 kDa) is also detected in every transfection. Triplicate samples were analyzed for each transfection. B95-8 and AG876: positive controls for expression of EBNA-2 type 1 (85 kDa) and type 2 (75 kDa), respectively; E2.5 + est: non-transfected EREB2.5 cells normally grown in medium supplemented with oestrogen. Immunoblotting for β-actin was performed as a loading control.

We previously developed a functional assay that distinguishes the ability of type 1 and type 2 EBNA-2 to maintain B cell proliferation using the EREB2.5 cell line [26], [86]. EREB2.5 cells contain a P3HR1 EBV genome lacking the EBNA-2 locus and a separate plasmid (p554-4) expressing the ER-EBNA-2 fusion protein. Hence, EBNA-2 activity and therefore survival and proliferation of the cells are dependent on oestrogen. In the absence of oestrogen, EBNA-2 function is inhibited by retention of the conditional ER-EBNA-2 protein in the cytoplasm but cell proliferation can be rescued if an OriP plasmid expressing constitutive type 1 EBNA-2 protein is transfected into the cells. In this situation the wild-type type 1 EBNA-2 functionally replaces the ER-EBNA-2 fusion protein. In contrast, if a wild-type type 2 EBNA-2-expressing OriP vector is transfected into oestrogen-starved cells, the cells undergo growth arrest. In this assay only type 1 but not type 2 constitutive EBNA-2 protein can provide essential EBNA-2 functions *in trans*. The *trans*-complementation assay was used in the present study to identify the regions of the type 1 EBNA-2 protein that can complement the deficiency of the type 2 protein to maintain proliferation of LCLs. For this, several type 1/type 2 EBNA-2 chimaeras were generated (by swapping sequences between the two types of protein) and these were tested for their ability to sustain cell growth of oestrogen-starved EREB2.5 cells. The complete set of chimaeras tested and the respective growth phenotype observed in the *trans*-complementation assay are shown in Figure 4 B. The chimaeras were precise swaps of EBNA-2 sequence without extraneous amino acids at the point of fusion. OriP-p294 plasmids expressing the chimaeric or wild-type type 1 or type 2 EBNA-2 proteins were Amaxa-transfected into EREB2.5 cells that had been growing normally in the presence of oestrogen. Immediately after transfection, oestrogen was withdrawn from the culture medium and cell proliferation was monitored over time by counting numbers of live cells (Figure 4 C). When the C-terminal region of type 1 EBNA-2 protein spanning from the WWPP motif (codons 323–326), within the RBP-Jk binding domain, to the end of the protein was swapped at homologous amino acid positions into the type 2 EBNA-2 protein (chimaera 2), live cell counts were similar to those obtained with wild-type type 1 EBNA-2 in the EREB2.5 growth assay (Figure 4 C). However, a null growth phenotype, similar to wild-type type 2 or empty vector control, was observed with the converse chimaera (chimaera 1), generated by joining together the type 1 sequence from codon 1 to the WWPP motif with type 2 sequences spanning from the WWPP motif to the last codon of the protein (Figure 4 C). Therefore, the C-terminus of type 1 EBNA-2 is able to confer a type 1 growth phenotype to type 2 EBNA-2 in the EREB2.5 *trans*-complementation assay. The C-terminus of the EBNA-2 protein includes the RG motif, the conserved region 7 (CR7), the transactivation domain (TAD) and the nuclear localization signal (NLS) (Figure 4 A). To further map the minimum type 1 sequences within the C-terminus of EBNA-2 responsible for the higher B cell immortalization efficiency, additional chimaeras were constructed by swapping different combinations of the RG, CR7, TAD and NLS regions from the type 1 into the type 2 protein (chimaeras 3 to 7). Only when the type 2 RG, CR7 and TAD regions were together replaced with homologous type 1 sequences (chimaera 7), accumulation of proliferating cells was restored to almost wild-type type 1 levels (Figure 4 C). In contrast, chimaeras from 3 to 6 behaved like wild-type type 2 EBNA-2, as they were unable to sustain proliferation (Figure 4 C). Long-term culture of oestrogen-independent EREB2.5 cells expressing the different EBNA-2 chimaeras and wild-type proteins resulted in establishment of continuously proliferating LCLs only in the case of chimaeras 2 and 7 and wild-type type 1 EBNA-2. These results demonstrate that, within the C-terminus of the type 1 EBNA-2, the RG, CR7 and TAD are the minimum type 1 sequences required to complement the lack of ability of type 2 EBNA-2 to rescue growth of oestrogen-depleted EREB2.5 cells.

To ensure that the lack of growth of oestrogen-depleted EREB2.5 cells was not due to either lack of expression or inappropriate protein localization of the chimaeras, western blot and immunofluorescence experiments were performed. Western blot analysis for EBNA-2 on extracts from transfected EREB2.5 cells grown without oestrogen revealed that all the chimaeric proteins display the expected size and are expressed at equal levels, similar to wild-type type 1 and type 2 EBNA-2 proteins (Figure 4 D). Levels of EBNA-2 expressed from the OriP plasmids was similar to those normally found in LCLs (Figure 4 D). For the analysis, the PE2 antibody was used, which was previously shown to detect the type 1 and the type 2 EBNA-2 with equal efficiency on a western blot [26]. Immunofluorescence analysis of HeLa cells transiently transfected with OriP-p294 plasmids, expressing the chimaeric and wild-type EBNA-2 proteins, confirmed that all the chimaeras are appropriately localized to the nucleus of the cells with exclusion from nucleoli (Figure S5A), with a pattern similar to that of wild-type type 1 and type 2 EBNA-2 (Figure S5B and [99]).

**Figure 5:**
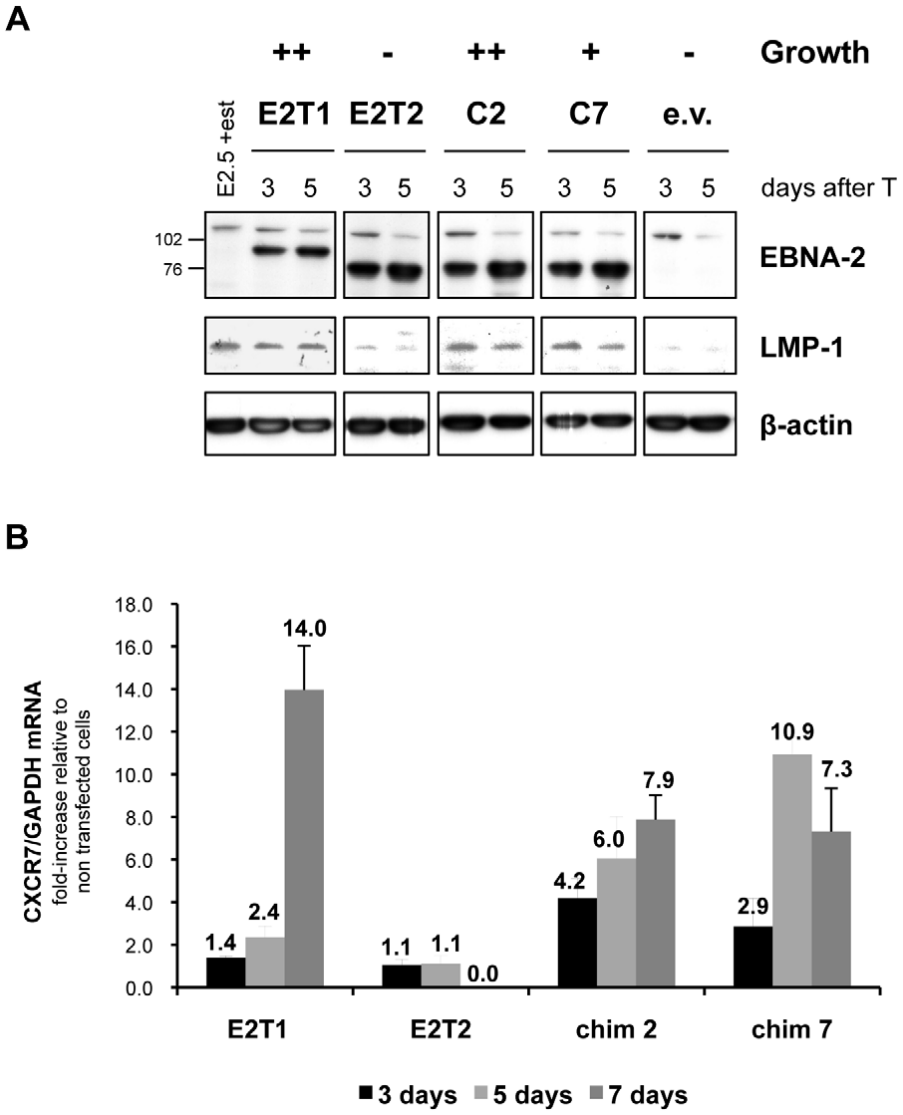
LMP-1 and CXCR7 expression induced by type 1 and type 2 EBNA-2 and chimaeras 2 and 7 correlates with the growth phenotype in the EREB2.5 growth assay. 6×10^6^ EREB2.5 cells were transfected with OriP-p294 plasmids expressing type 1 EBNA-2 (E2T1), type 2 EBNA-2 (E2T1), chimaera 2 (C2) and chimaera 7 (C7) or with an empty vector (e.v.) and grown in the absence of oestrogen. At 3, 5 (**A**) and 7 (**B**) days after transfection (T), cells were harvested and lysed in either RIPA lysis buffer to extract proteins (**A**) or in Trizol (Invitrogen), to extract total RNA (**B**). (**A**) Protein samples were subjected to western blot analysis, probing for EBNA-2 (PE2 antibody), LMP-1 (CS 1-4 antibody) and β-actin, as loading control. Constitutive EBNA-2 proteins, wild-type type 1 (85 kDa), type 2 and chimaeric (75 kDa), as well as the conditional ER-EBNA-2 (120 kDa) are detected with the PE2 antibody. Numbers alongside the EBNA-2 immunoblot panel represent the apparent molecular weight (in kDa). (**B**) Total cell RNA samples were retro-transcribed into cDNA and analyzed by qPCR to quantify the levels of CXCR7 transcripts. No RNA could be extracted from type 2 EBNA-2-transfected cells at the day 7 time-point, since the majority of the cells were dead (> 70% relative to type 1 EBNA-2-transfected cells). The histograms show, for each transfection, the ratio CXCR7/GAPDH mRNA relative to non-transfected EREB2.5 cells grown in the presence of oestrogen. The analysis was performed in duplicate. Error bars represent standard errors. Data from 1 representative experiment of 2 is shown.

Expression of the EBNA-2-direct viral target and major EBV oncogene LMP-1 was assessed in the EREB2.5 growth assay. Western blot analysis revealed that at 3 and 5 days after transfection, in oestrogen-starved cells expressing type 1 EBNA-2 and chimaeras 2 and 7, LMP-1 protein expression was maintained at levels similar to those detected in EREB2.5 cells normally grown in the presence of oestrogen (Figure 5 A). In contrast, LMP-1 expression was lost in type 2 EBNA-2 or empty vector-transfected cells (Figure 5 A). qRT-PCR analysis for the mRNA of the cell gene CXCR7 showed increased levels following expression of either type 1 EBNA-2 or chimaeras 2 and 7, with a 14-fold increase (type 1 EBNA-2) or approximately 7-fold increase (chimaeras 2 and 7), compared to non-transfected cells, at 1 week after transfection (Figure 5 B). In contrast, when type 2 EBNA-2-expressing vector was transfected, CXCR7 mRNA was maintained at the same levels normally observed in non-transfected cells (fold-increase of 1) up to 5 days after transfection but after this time-point it was not further induced and the cells died (Figure 5 B). These results demonstrate that LMP-1 protein and CXCR7 mRNA expression levels, induced by type 1 and type 2 EBNA-2 and chimaeras 2 and 7, correlate with the growth phenotype in the EREB2.5 *trans*-complementation system. Loss of LMP-1 and CXCR7 expression in type 2 EBNA-2-expressing cells is rapidly followed by growth arrest of the cells, whereas in cells expressing type 1 EBNA-2, chimaera 2 or 7, maintenance of LMP-1 and CXCR7 expression is accompanied by sustained proliferation of the cells. Therefore the C-terminus of type 1 EBNA-2 in chimaeras 2 and 7 is sufficient to confer induction of type 1 growth phenotype and approximately type 1 expression levels of LMP-1 and CXCR7.

### Functional differences between EBNA-2 types, mapped to the C-terminus, are typical of type sequence

In our mapping analysis performed to identify the type 1 regions of EBNA-2 important for B cell growth, EBNA-2 sequences from the EBV strains B95-8 (type 1 prototype) and AG876 (type 2 prototype) were used. To ensure that the functional differences being mapped are typical of EBV type sequences, the C-terminus of EBNA-2 from additional type 1 and type 2 isolates was sequenced and compared to the prototypes B95-8 and AG876. The EBV strains chosen for the analysis were derived from laboratory LCL, BL and NPC cell lines (Table 1). Comparison of type 1 isolates to the type 1 strain of reference B95-8 showed that, in the region examined, there is a very high percentage of identity (> 98%) with only a few nucleotide sequence variations detected. Most of the changes are silent but a few produce a change in the amino acid coded. Changes in the amino acid sequence were almost always located outside the known EBNA-2 functional domains; only in 2 strains (Mak 1 and MABA, Table 1) were there changes within a functional domain (TAD and NLS respectively). Similarly, alignment of the sequence coding for the C-terminus of EBNA-2 from several type 2 isolates against the prototype strain AG876, indicated that the sequence identity is almost 100%, with only 2 strains (Alouek and Wewak, Table 1) displaying nucleotide variations (2 silent and 1 non-silent, but localized outside functional domains). These results indicate that the functional differences between type 1 and type 2 EBNA-2, which we have mapped to this region, are representative of viral type sequence.

**Table 1:** Sequence analysis of the C-terminus of EBNA-2 from type 1 and type 2 EBV isolates obtained from laboratory LCL, BL and NPC cell lines.

Cell line	EBV type	Sequence identity compared to B95-8^1^	Sequence identity compared to AG876^2^	Silent nucleotide variations	non-silent nucleotide variations outside functional domains	non-silent nucleotide variations within functional domains
JAC-B2	1	100%	∼76%	0	0	0
Akata	1	99.8%	∼76%	1	0	0
Raji	1	99.8%	∼76%	1	0	0
C2+Obaji	1	98.8%	∼76%	3	3	0
C666.1	1	99%	∼76%	3	2	0
Mak 1	1	98.6%	∼76%	3	3	1 (Ala452Thr, within TAD^3^)
MABA	1	99.2%	∼76%	2	1	1 (Pro478Ser, within NLS^4^)
SI-B1	1	100%	∼76%	0	0	0
AF-B1	2	∼76%	100%	0	0	0
WEI-B1	2	∼76%	100%	0	0	0
Alouek	2	∼76%	99.8%	1	0	0
C2+BL16	2	∼76%	100%	0	0	0
Jijoye	2	∼76%	100%	0	0	0
Wewak	2	∼76%	99.6%	1	1	0

The laboratory cell lines analysed include: LCLs (B95-8, JAC-B2, C2+Obaji, MABA, AF-B1, WEI-B1, Aloueck, C2+BL16), one NPC (C666.1) and BLs (Akata, Raji, Mak 1, SI-B1, AG876, Jijoye, Wewak).

1 Genbank accession number AJ507799;

2 Genbank accession number DQ279927;

3 TAD: transactivation domain;

4 NLS: nuclear localization signal.

### Cooperation between type 1 or type 2 EBNA-2 and EBNA-LP in the EREB2.5 growth system

**Figure 6:**
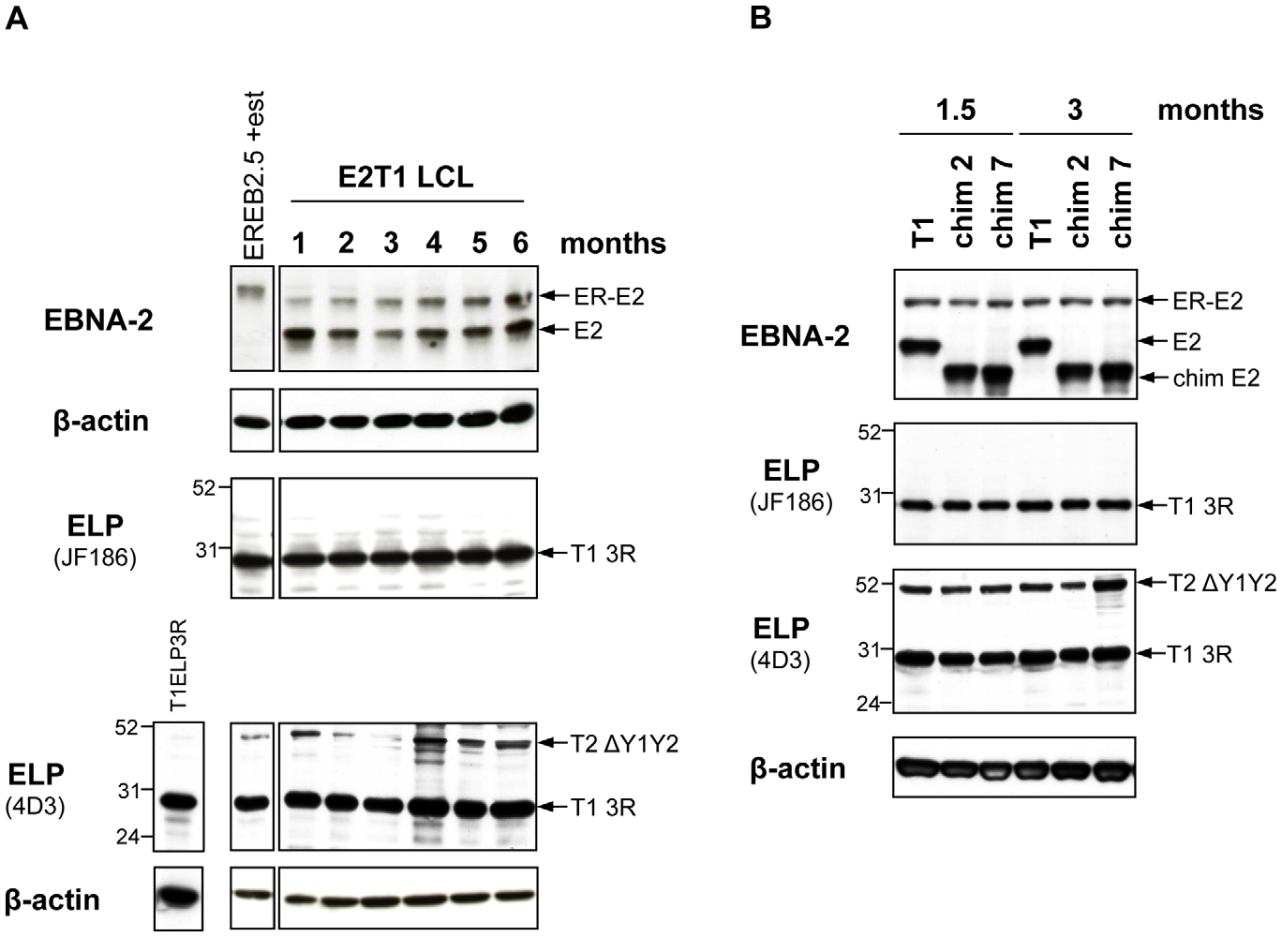
Characterization of oestrogen-independent LCLs established from EREB2.5 cells expressing chimaeras 2 and 7. EREB2.5 cells transfected with OriP-p294 plasmids coding for type 1 EBNA-2 (**A**) or chimaeras 2 and 7 (**B**) and grown in the absence of oestrogen gave rise to continuously proliferating LCLs. Protein samples were harvested from these LCLs at each month after transfection over a period of 6 months (**A**) or after 1.5 and 3 months (**B**) and analyzed by western blotting. The anti-EBNA-2 antibody (PE2), detected the transfected type 1 (E2) and chimaeric (chim E2) EBNA-2 proteins as well as the endogenous ER-EBNA-2 (ER-E2). EBNA-LP immunoblotting was performed with the antibodies JF186 (type 1-specific) and 4D3 (recognizes both types). At any time-point analyzed, both antibodies detected a type 1 3-repeat EBNA-LP species (T1 3R), expressed from the endogenous p554-4 plasmid, indicating that the plasmid was not lost from the cells. The number of repeats was judged by comparison to 293 cells transfected with a type 1 3-repeat EBNA-LP-expressing vector (T1ELP3R). A type 2 Y1Y2-truncated isoform of around 52 kDa (T2 ΔY1Y2), expressed by the resident P3HR1 genome, was also detected by the 4D3 antibody. Numbers alongside the EBNA-LP immunoblot panel represent protein molecular weight (in kDa). β-actin immunoblots ensure equal loading of the proteins.

EBNA-2-mediated activation of both cell (cyclin D2) and viral promoters (LMP-1/LMP-2B and Cp) can be greatly augmented by cooperation with the EBV latent protein EBNA-LP, which has been shown to be required for optimal virus-induced B cell transformation [9], [27]–[29]. Several EBNA-LP proteins, of different type and size, are believed to be present in the EREB2.5 *trans*-complementation system. To address whether these EBNA-LP species may influence the growth phenotype induced by the wild-type (type 1 and type 2) and chimaeric EBNA-2 proteins in oestrogen-starved EREB2.5 cells we first determined which specific EBNA-LP isoforms are expressed in the EREB2.5 cell line and in the derivative oestrogen-independent LCLs established with type 1 EBNA-2 and chimaeras 2 and 7 (Figure 6). For this, western blot analysis was performed comparing two anti-EBNA-LP antibodies: JF186 (type 1-specific) and 4D3, which recognizes both types of EBNA-LP [89], [92]. Using the 4D3 antibody, a type 2 EBNA-LP species of around 52 kDa was detected in non-transfected EREB2.5 cells, normally grown in oestrogen-supplemented medium (Figure 6 A, lane EREB2.5 + est), and in all the oestrogen-autonomous LCLs (Figure 6 A and B). This isoform is encoded by the resident P3HR1 genome and is known to lack the Y1 and Y2 domains [100]. Both antibodies detected a type 1 EBNA-LP isoform containing 3 W repeats, as determined by comparison with lysates from 293 cells transiently transfected with a plasmid expressing 3-repeat EBNA-LP (Figure 6 A, lane T1ELP3R). This EBNA-LP is encoded by the endogenous plasmid p554-4 and it was detected not only in non-transfected oestrogen-dependent EREB2.5 cells (Figure 6 A, lane EREB2.5 + est), but also in all the oestrogen-independent LCLs established by transfecting chimaeras 2 and 7 or type 1 EBNA-2, analyzed over a period of 6 and 3 months (Figure 6 A and B, respectively). This observation suggests that in these experiments the p554-4 plasmid is not completely lost from the oestrogen-free LCLs. This was corroborated by the EBNA-2 immunoblot that showed continuous expression of ER-EBNA-2 over time, although at variable levels. It is likely that some of the p554-4 plasmid has become integrated in these cells since, after extraction of low molecular weight DNA from the oestrogen-independent LCLs, only the OriP-p294 plasmid (expressing constitutive EBNA-2 proteins) but not the p554-4 vector was rescued (data not shown).

**Figure 7:**
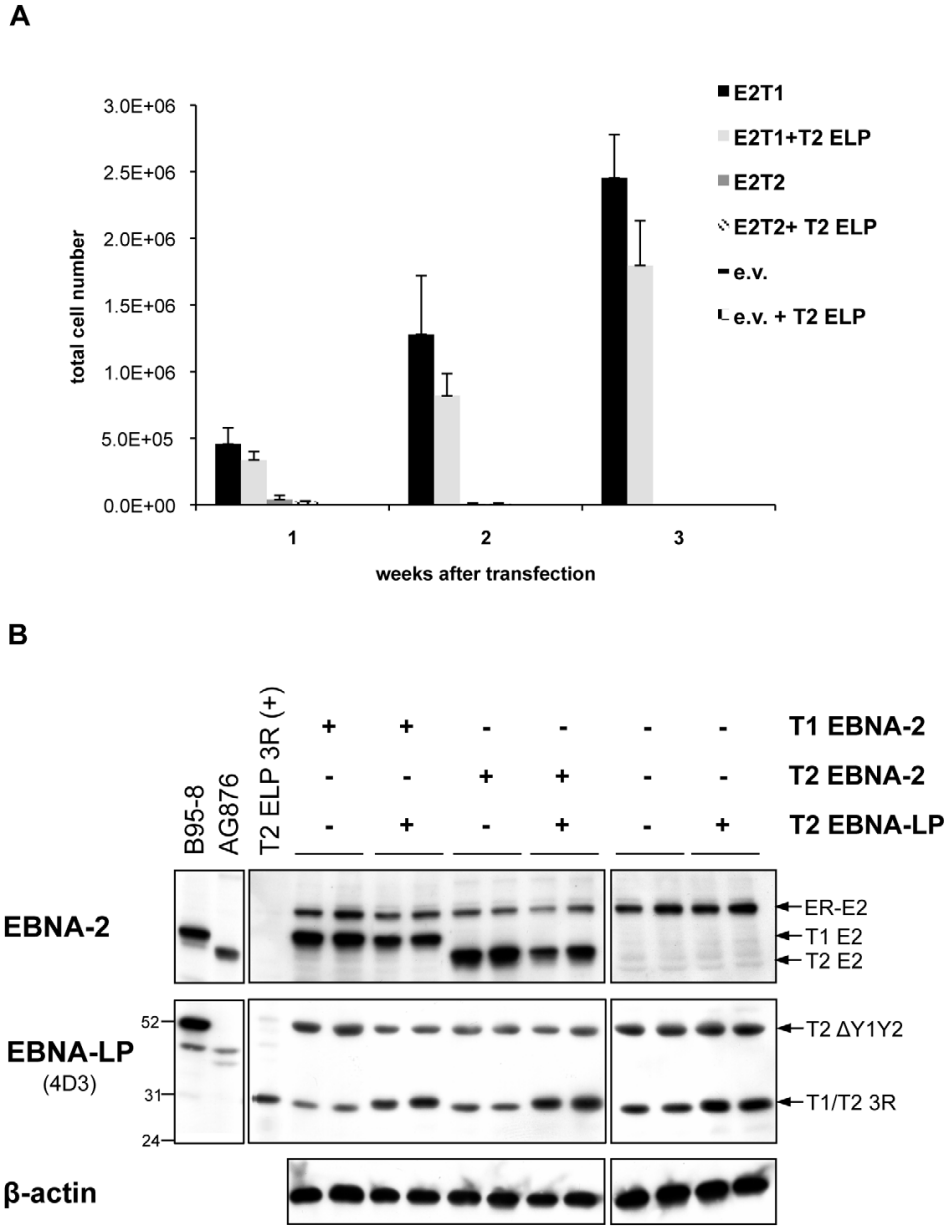
Cooperation between type 1 and type 2 EBNA-2 and EBNA-LP in the EREB2.5 growth system: The assay functionally distinguishes between the ability of type 1 and type 2 EBNA-2 to sustain cell proliferation, regardless the type of EBNA-LP. (**A**) 6×10^6^ EREB2.5 cells were transfected with OriP-p294 plasmids expressing either type 1 (E2T1) or type 2 (E2T2) EBNA-2, or with empty vector (e.v.), in the presence or absence of a pSNOC plasmid expressing a type 2 EBNA-LP with 3 repeats (T2 ELP). Oestrogen was removed immediately after transfection and outgrowth of the cells was monitored over a period of 3 weeks. Transfections were performed in duplicate and for each replica, triplicate wells of cells were counted. Error bars indicate standard deviations. (**B**) 4 days after transfection, proteins were extracted from the cells and fractionated by SDS-PAGE. Western blot analysis was performed using PE2 and 4D3 antibodies in order to detect EBNA-2 and EBNA-LP respectively and confirmed equal levels of expression. The PE2 antibody, detected the transfected type 1 (T1 E2) and type 2 (T2 E2) EBNA-2 proteins as well as the endogenous ER-EBNA-2 (ER-E2). EBNA-LP western blot showed that empty vector-transfected cells express the 30 kDa type 1 full-length 3-repeat EBNA-LP (T1 3R), encoded by the p554-4 plasmid, and the 52 kDa Y1Y2-truncated EBNA-LP species (T2 ΔY1Y2), expressed from the P3HR1 resident genome. The full-length type 2 3-repeat EBNA-LP protein (T2 3R), expressed from the pSNOC vector, is detected on the gel at approximately the same position of the endogenous type 1 3-repeat EBNA-LP (see main text for details). The levels of β-actin did not alter throughout the experiment. Duplicate samples for each transfection are shown. B95-8, AG876 and T2 ELP 3R (+) lanes represent positive controls for expression of EBNA-2, type 1 and type 2, and type 2 3-repeat EBNA-LP, respectively. Numbers indicate apparent molecular weight (in kDa).

Since only a type 1 full-length EBNA-LP is present in the EREB2.5 assay, it might be that a type-specific cooperation between EBNA-2 and EBNA-LP would be important for optimal proliferation in the EREB2.5 growth system and that the lack of a full-length type 2 EBNA-LP species could hamper the growth-promoting abilities of type 2 EBNA-2. We tested this issue directly by transfecting plasmids expressing either type 1 or type 2 EBNA-2 into EREB2.5 cells, in the presence or absence of a plasmid expressing a 3-repeat type 2 EBNA-LP, to check whether the presence of a full-length type 2 EBNA-LP (homologous to the endogenous type 1 3-repeat EBNA-LP) would allow the type 2 EBNA-2 to sustain cell proliferation in an oestrogen-independent fashion (Figure 7). Accumulation of proliferating cells was assessed each week after transfection and oestrogen withdrawal over a period of 3 weeks (Figure 7 A). Cells expressing type 1 or type 2 EBNA-2 alone displayed proliferation levels similar to those observed in Figure 4 C. Addition of the type 2 EBNA-LP did not produce any significant effect on growth of either type 1 or type 2 EBNA-2-expressing cells or control cells. There were no significant differences in the levels of EBNA-2 proteins comparing the type 1 and type 2 forms, as assessed by western blotting with the PE2 antibody (Figure 7 B). Immunoblotting with the 4D3 antibody confirmed good expression of the type 2 3-repeat EBNA-LP from the transfected pSNOC vector. This EBNA-LP isoform displays a slightly higher molecular weight on SDS-PAGE compared to the endogenous type 1 3-repeat EBNA-LP (Figure 7 B) because of the substantial differences (12 amino acids) in the primary amino acid sequences between type 1 and type 2 EBNA-LPs. This experiment demonstrates that the inability of type 2 EBNA-2 to rescue growth of oestrogen-depleted EREB2.5 cells is not due to the absence of a full-length type 2 EBNA-LP in the system.

## Discussion

The most striking biological difference between type 1 and type 2 EBV is that type 1 strains are far better than type 2 at inducing primary B lymphocyte transformation *in vitro* and the EBNA-2 gene is the key determinant of this difference [5], [19]. Following EBV infection of *naïve* B cells *in vitro*, EBNA-2 drives B cell transformation into LCLs by acting as a potent transcriptional activator of both viral and cell genes which in turn will then cause survival and proliferation of the infected cells [1], [23]–[26]. Our results demonstrate a mechanism that can account for the superior ability of type 1 EBV to produce LCLs. Primary B cell infection experiments using BAC-derived recombinant EB viruses showed that higher and more rapid induction of some EBNA-2-target genes (which are required for continuous proliferation of EBV-infected LCLs) by type 1 EBNA-2-expressing virus correlates with the enhanced B cell transforming capability of this virus (Figure 3 and Figure S3). In this study we have focussed our analysis on two genes, among all the direct target genes of EBNA-2, based on their crucial importance in B cell proliferation in the context of EBV infection and these are the viral oncogene LMP-1 and the cell gene CXCR7. Among the viral EBNA-2-targets, LMP-1 is the major EBV oncogene and is required for initiation of primary B cell transformation *in vitro* [8] and also for maintenance of continuous proliferation of EBV-LCLs [62]. Our time-course analysis of induction of LMP-1 and CXCR7 during the initial stages of infection with type 1 or type 2 EBNA-2 BAC-EBV, revealed that with type 2 EBNA-2 LMP-1 induction is delayed and weaker compared to type 1 EBV-infected cells, and CXCR7 expression levels are not maintained consistently throughout the time-points analyzed with type 2 EBNA-2 (Figure 3 B and C). However, once a type 2 LCL is eventually established (up to 4 months after infection, with a delay of approximately 3 months compared to type 1 LCLs), LMP-1 and CXCR7 expression levels are similar to those detected in type 1 LCLs (Figure 3 B and C), confirming that those genes are essential for long-term proliferation of EBV-LCLs. Our results indicate that the key difference in transformation efficiency between type 1 and type 2 EBV is determined by differences in induction of the pro-survival genes LMP-1 and CXCR7 occurring during the early stages of infection. Differential regulation of LMP-1 and CXCR7 by the two types of EBNA-2-expressing viruses during infection is consistent with the results obtained in cell line based assays. We have shown that in Daudi and P3HR1 cl.16 cell line backgrounds, using oestrogen-regulated EBNA-2 proteins, LMP-1 protein and CXCR7 mRNA are more potently and rapidly induced by the type 1 EBNA-2 (Figure 1 and [26]).

Our finding that the CXCR7 gene, together with LMP-1, accounts for the greater transforming potential of type 1 EBV, compared to type 2, is particularly relevant in light of the fact that two other oncogenic viruses, Kaposi's Sacroma-associated herpesvirus (KSHV) and human T-lymphotropic virus type 1 (HTLV-1), have been shown to up-regulate this gene during infection. CXCR7 induction by HTLV-1 Tax protein has been reported to have a pro-survival effect on HTLV-1-infected T cells and in KSHV-infected hDMVECs induction of CXCR7 is essential for spindle cell transformation, much as occurs with EBV-mediated B cell transformation [78], [79], [101]-[103]. Targeting of CXCR7 may therefore represent a common mechanism exploited by oncogenic viruses to induce cell transformation.

CXCR7 binds to the same ligand as CXCR4 (CXCL12/SDF-1) and has been proposed to function as a modulator of CXCR4-mediated signalling [77], [104], [105]. Several reports indicate that CXCR4 is down-regulated in primary B cells upon infection by EBV [95]–[97] and this was confirmed by our qRT-PCR time-course analysis during infection with type 1 and type 2 EBNA-2 recombinant viruses (Figure 3 D). At least five EBV latent proteins have been found potentially to be involved in modulation of CXCR4 expression in EBV-LCLs. Stable expression of EBNA-2 and LMP-1 in the EBV-negative BJAB cell line was shown to induce down-regulation of CXCR4 [96]. Moreover, whole genome microarray studies indicated that CXCR4 expression is down-regulated by EBNA-3A and EBNA-3B in EBNA-3A and -3B knock-out LCLs, but up-regulated by EBNA-3C in LCLs with conditional EBNA-3C protein [106]–[109]. In our infection experiments CXCR4 was effectively repressed in both type 1 and type 2 EBNA-2-virus infected cells by the day 8 time-point (Figure 3 D). In both type 1 and type 2 infections, the kinetics of CXCR4 regulation were almost complementary to that of LMP-1 (Figure 3 B and D), consistent with the notion that both EBNA-2 and LMP-1 contribute to but are not the only determinants of CXCR4 regulation.

From the EBNA-2 chimaeras tested, chimaeras 2 and 7 indicated that the RG, CR7 and TAD are the minimum type 1 sequences required to confer a type 1 growth phenotype to a type 2 EBNA-2 protein in the EREB2.5 *trans*-complementation assay (Figure 4 B and C). This result was quite surprising and unexpected as most of the sequence variation between type 1 and type 2 EBNA-2 lies in the N-terminal half of the protein, mostly in the diversity region (Figure 4 A) and not in the C-terminus. In fact, in the C-terminus the sequence homology between the two types of EBNA-2 proteins is 62%, whereas in the N-terminus this is 48% and only 30% in the diversity region. Importantly, in the EREB2.5 assay the growth phenotype correlated with the ability of the EBNA-2 proteins to induce LMP-1 and CXCR7 genes. Type 1 EBNA-2 and chimaeras 2 and 7 maintained high levels of expression of those genes, which in turn drive continuous proliferation of the cells leading to LCLs establishment (Figures 5 and 6), whereas in cells expressing type 2 EBNA-2, LMP-1 and CXCR7 were not maintained at normal physiological levels (Figure 5). These results show that the C-terminus of type 1 EBNA-2 protein confers the ability to give sufficient expression levels of those genes that are essential for EBV-LCL proliferation (LMP-1, CXCR7). In summary, the data indicate that the mechanism that accounts for the superior ability of type 1 EBNA-2 to promote B cell growth, compared to the type 2, is higher induction of LMP-1 and CXCR7 mediated by the C-terminal part of the protein. Although the differential effects on LMP-1 and CXCR7 would be sufficient to cause the reduced transformation by type 2 EBV, we have not shown that these are the only genes involved. Attempts to complement the deficiency in transformation by co-transfecting LMP-1 and CXCR7 expression plasmids in the EREB2.5 growth assay were not successful (data not shown). However, the technical difficulty of maintaining the correct level of all the plasmids in the cells (EBV, p554-4, LMP-1 and CXCR7 expression vectors) may well account for that result.

The TAD of EBNA-2 is a strong transcriptional activator and is essential for EBNA-2-mediated activation of viral and cell genes and B cell transformation, since viruses lacking this domain are immortalization-incompetent [38], [65]. The TAD mediates transcriptional activation at EBNA-2-target promoters by recruiting histone acetyltransferases [54] and several host transcription factors [51]–[53]. The RG sequence is involved in protein-protein and protein-nucleic acid interactions and is important for efficient B cell growth transformation [44]. The RG element down-regulates EBNA-2 activation of the LMP-1 promoter but not of the Cp [44]. The CR7 has been shown to be dispensable for B cell growth transformation, although is important for transcriptional cooperation with EBNA-LP [44]. In addition to the CR7, the RG and TAD domains have also been shown to be involved in EBNA-2/EBNA-LP transcriptional cooperation. In fact, the TAD of EBNA-2 has been demonstrated to be required for EBNA-LP co-activation with EBNA-2 in the context of LMP-1-promoter reporter assays and represents a specific binding site for EBNA-LP, as demonstrated by *in vitro* binding assays [31]. Moreover, the RG and CR7 elements are able to down-regulate the high intrinsic transcriptional activity of EBNA-2 TAD in the context of Gal4 DNA-binding fusions, suggesting a modulatory activity of these domains on EBNA-LP function [37]. EBNA-LP has been shown to enhance EBNA-2-mediated transcriptional activation of both viral and cell promoters (LMP-1/LMP-2B, Cp and cyclin D2) [27]–[31]; the mechanism of cooperative function is not entirely clear but has been proposed to involve interaction with histone deacetylase 4 [110]. EBNA-2 and EBNA-LP proteins do not significantly associate in lymphoblasts, as demonstrated by co-immunoprecipitation and immunofluorescence assays in previous studies [30], [31] and it has therefore been suggested that EBNA-LP co-activation with EBNA-2 takes place only when transient unstable interactions are established, which would allow recruitment of positive transcriptional regulators to the EBNA-2 TAD at EBNA-2-specific promoters [31].

Our mapping studies suggest that the difference in LMP-1/CXCR7 activation and therefore in growth phenotype observed between type 1 and type 2 EBNA-2 is determined by different mechanisms of transcriptional activation, mediated by the TAD of EBNA-2 and modulated by cooperation with EBNA-LP, through the RG, CR7 and TAD EBNA-2 elements. Our results are consistent with the crucial roles played by the EBNA-2 TAD-mediated transcriptional activation and the EBNA-LP cooperative function during the EBV-driven B cell transformation process [28], [29], [38]. Ongoing additional detailed mutational analysis of the RG, CR7 and TAD sequences may identify the amino acids that are the key determinants of the differing abilities of type 1 and type 2 EBNA-2 to transactivate target genes, and therefore sustain B cell growth. At the biochemical level the differences between type 1 and type 2 transcriptional activation may be determined by differences in cell transcription factors or histone acetyltransferases recruited by type 1 EBNA-2 TAD, compared to the type 2, resulting in higher induction of LMP-1 and CXCR7. It is noteworthy in this context that EBNA-2 regulation of the LMP-1 promoter occurs mainly through other cell transcription factors such as PU.1, rather than RBP-Jk [46]–[49], [54], [63]. It will be interesting to determine in the future whether a similar situation exists at the CXCR7 promoter. We envisage the most likely mechanism for the differential regulation of LMP-1 and CXCR7 compared to other EBNA-2 target genes would be that their promoters may both require specific transcription factors that cooperate with EBNA-2, transcription factors not required for most EBNA-2-responsive genes. We are currently investigating the CXCR7 promoter that is active in EBV-LCLs to test this hypothesis.

Bearing in mind the importance of EBNA-LP for enhancement of the ability of EBNA-2 to activate genes essential for proliferation, such as LMP-1 [28], [29], we determined which EBNA-LP species are expressed in the EREB2.5 assay and detected a type 1 full-length EBNA-LP and a type 2 Y1Y2-truncated isoform (Figure 6). Even though the Y1Y2 region has been shown to have only a modulatory effect on EBNA-LP cooperative function [28], [29], one could speculate that the type 1 full-length EBNA-LP might cooperate more efficiently with the type 1 EBNA-2 than with the type 2 EBNA-2, thereby determining the null growth phenotype of the latter EBNA-2 type in the assay. However, the experiment illustrated in Figure 7 clearly demonstrates that this is not the case, since even in the presence of a full-length type 2 EBNA-LP, the inability of type 2 EBNA-2 at maintaining cell proliferation was not complemented. Therefore the EREB2.5 growth assay functionally distinguishes between the ability of type 1 and type 2 EBNA-2 to sustain cell proliferation, which is likely to be enhanced by cooperation with EBNA-LP but is not influenced by the type of EBNA-LP present in the system. A role in protection from caspase-induced apoptosis has been recently proposed for the Y1Y2-truncated type 2 EBNA-LP species in BL cell lines [88], however the possible contributions of this additional function of the EBNA-LP mutant to our EREB2.5 growth assay have not yet been explored.

Likewise, in the Daudi and P3HR1 cl.16 systems with oestrogen-inducible EBNA-2 proteins used to analyse the time-course induction of LMP-1 and CXCR7 by type 1 and type 2 EBNA-2 (Figure 1), several full-length type 1 EBNA-LP isoforms are expressed from the ER-EBNA-2-coding vectors, in addition to the type 2 Y1Y2-truncated species, encoded by the endogenous EBV genomes (Figures S1 and S2). Similarly to the EREB2.5 assay, it might be speculated that the absence of a full-length type 2 EBNA-LP could account for the weaker induction of LMP-1/CXCR7 by type 2 EBNA-2. By using a functional assay for EBNA-LP cooperative function [17], [29] based on the Daudi cell line, we demonstrated that this is not the case (Figure 2). Co-expression of type 2 EBNA-2 with either type 1 or type 2 full-length EBNA-LP in the Daudi cell line induced only a weak up-regulation of the endogenous LMP-1 gene, which was markedly lower than that observed when type 1 EBNA-2 was co-expressed with either type 1 or type 2 EBNA-LP. Therefore the weaker induction of LMP-1 by type 2 EBNA-2 is not affected by the EBNA-LP type. In all our assays for EBNA-LP cooperative function an EBNA-LP construct with 3 repeat W1W2 domains has been used, which is above the minimum (2 repeats) required for optimal EBNA-LP function [17], [29], [60].

Our infection assays are similar to the EREB2.5 system and to the Daudi/P3HR1 cl.16:ER-EBNA-2 systems in that both the type 1 and the type 2 EBNA-2 BAC viruses express full-length type 1 EBNA-LP species (Figure S4), containing at least 3 W1W2 repeats. Based on the results discussed before and illustrated in Figures 2 and 7, showing that the weaker induction of LMP-1 in Daudi and the inability to maintain long-term growth in the EREB2.5 assay by type 2 EBNA-2 is not affected by the type of EBNA-LP present, we can infer that the gene-expression pattern (Figure 3) and the growth phenotype (Figure S3) induced by type 2 EBNA-2-expressing BAC virus in the infection experiments is not affected by the absence of a type 2 EBNA-LP. Moreover, the low-efficiency transformation phenotype produced by our type 2 EBNA-2 BAC recombinant EBV is similar to that reported for the wild-type type 2 AG876 EBV strain virus [19].

Our finding that type 2 EBNA-2 cannot rescue proliferation of oestrogen-starved EREB2.5 cells when expressed in *trans* in the EREB2.5 assay (Figure 4 B and C) is somehow surprising, if one considers that a type 2 EBV virus is able to eventually establish LCLs upon infection of primary B cells, albeit with very low efficiency (Figures 3 and S3 and [19]). This difference might be either ascribed to the truncated EBNA-LP protein present in the EREB2.5 assay, or, more likely, to additional factors linked to the virus/B cell interaction occurring during the process of infection. These events occurring during the early stages of infection might act as compensatory mechanisms for the poor ability of type 2 EBNA-2 to induce B cell proliferation. LCL outgrowth following initial EBV infection of *naïve* resting B cells is a stochastic event whereby a few cells with the correct levels of expression of those genes required for B cell proliferation (e.g. CXCR7, LMP-1) are selected and clonally expanded. It seems likely that slower and lower expression of LMP-1/CXCR7 induced by type 2 EBNA-2-expressing virus during the initial stages of infection accounts for the low transforming efficiency displayed by this viral type. However, once a few cells infected with type 2 EBV eventually express the correct levels of LMP-1/CXR7, these cells are selected and can be amplified to give rise to an LCL. In fact, in both type 1 and type 2 established LCLs, LMP-1 and CXCR7 expression levels were ultimately approximately similar (Figure 3 B and C and [26]).

In this study we have identified a mechanism that accounts for a very remarkable biological difference between type 1 and type 2 EBV, namely the poorer *in vitro* transforming ability of type 2 strains of EBV, compared to type 1. This mechanism consists of lower and delayed induction of both viral (LMP-1) and host (CXCR7) genes which are essential for continuous proliferation of EBV-infected LCLs and is mediated by the C-terminal region of the EBNA-2 protein.

## Materials and Methods

### Cell lines

P3HR1 clone 16, Daudi and Raji are EBV-positive BL cell lines [111]-[113]. B cell lines were maintained in RPMI 1640 medium (Gibco-BRL) supplemented with 10 to 20% (v/v) heat-inactivated foetal bovine serum (FBS, BioWhittaker) and antibiotics (100 units/ml penicillin and 100 units/ml streptomycin, Gibco-BRL). EREB2.5 cells [86] contain a conditional EBNA-2 protein regulated by oestrogen and were maintained in RPMI 1640 medium supplemented with 10% FBS, antibiotics and 1 µM β-oestradiol (Sigma). Daudi:ER-EBNA-2 T1/T2 [26] and P3HR1 cl.16:ER-EBNA-2 T1/T2 stable cell lines were grown in RPMI 1640 medium supplemented with 10% FBS, antibiotics and 400 or 500 µg/mL G418 (Calbiochem) respectively. 293 and HeLa cell lines were grown in Dulbecco Modified Eagle Medium (DMEM, Gibco-BRL) supplemented with 10% FBS and antibiotics. 293 cell lines stably transfected with EBV-BAC constructs were maintained in DMEM with 10% FBS, antibiotics and 100 µg/ml Hygromycin B (Roche).

### Plasmids

The OriP-p294 plasmids used for expression of EBNA-2, type 1 and type 2, and the EBNA-2 chimaeras, were derived from the CMVpEBNA-1 plasmid described in [114]. The EBNA-1 coding region of the CMVpEBNA-1 plasmid was extracted and replaced with a multiple-cloning site (MCS) comprising *Hind*III, *Bam*HI and *Not*I restriction sites, generating the OriP-p294 MCS plasmid. To generate the wild-type type 1 EBNA-2-expressing plasmid, the region comprising one BamHI W repeat and the type 1 EBNA-2 coding region from the p554 plasmid (kind gift from Bettina Kempkes, described in [86], [115]) was cloned as a *Bgl*II – *Not*I fragment into OriP-p294 MCS, between *Bam*HI and *Not*I sites. To generate the homologous vector expressing a type 2 EBNA-2, the fragment encompassing one BamHI W repeat and the type 2 EBNA-2 sequence from the pAG1 vector (described in [26]; this is essentially homologous to p554, but carries a type 2 EBNA-2) was extracted by *Bgl*II – *Not*I digestion and cloned between *Bam*HI and *Not*I sites in the OriP-p294 MCS vector. This procedure produced the two OriP-p294 plasmids with either type 1 or type 2 EBNA-2 sequences (OriP-p294 E2T1/T2). The expression of the EBNA-2 proteins from these plasmids is driven by the EBV Wp promoter. Additional features of these vectors include the EBV origin of replication, OriP, and a hygromycin-resistance gene.

To generate the EBNA-2 chimaeric sequences, two pBluescript plasmids carrying type 1 or type 2 EBNA-2 coding sequences between *Eco*RI and *Not*I sites, extracted from the p554 and pAG1 plasmids respectively [26], [86], were used. For chimaeras 1 and 2, a fragment spanning between two *Bst*XI sites (one in the EBNA-2 ORF, mapping to the WWPP amino acid motif, codons 323-326, within the RBP-Jk domain, and one in the vector backbone, downstream *Not*I site) was swapped between the two constructs. To generate chimaera 3, the region comprising the CR7, TAD and NLS elements from type 1 EBNA-2 was *Pfu*-PCR amplified using a primer with overhanging *Sac*II (5′) restriction site and the universal T3 primer. A *Sac*II restriction site was artificially inserted by site-directed mutagenesis, using the QuickChange Site-directed mutagenesis kit (Stratagene), in type 2 EBNA-2 sequence, without altering the amino acid sequence (codons 337-338, between the RG sequence and the CR7). The type 1 PCR amplicon was then cloned as a *Sac*II – *Not*I fragment at homologous positions in the type 2 EBNA-2 vector. A similar strategy was used to generate chimaera 4. Briefly, a PCR amplicon, consisting of the TAD and the NLS sequences of type 1 EBNA-2, was generated by *Pfu*-DNA polymerase PCR using an oligonucleotide carrying an overhanging *Eco*RV (5′) restriction site and the universal primer T3. This PCR product was then cloned as an *Eco*RV – *Not*I fragment into the pBluescript plasmid carrying type 2 EBNA-2 ORF, in which a silent *Eco*RV site had been previously introduced by *in vitro* mutagenesis at codons 388-389, between the CR7 and the TAD. To produce chimaera 5, the RG sequence from type 1 EBNA-2 was amplified by PCR with *Pfu* DNA polymerase using primers with overhanging *Bst*XI (5′) and *Sac*II (3′) sites and cloned between the same restriction sites into the type 2 EBNA-2 pBluescript plasmid, carrying the artificial *Sac*II site. Likewise, to make the chimaera 6 construct, the type 1 sequences encompassing the RG and the CR7 domains were *Pfu*-PCR amplified with primers bearing overhanging *Bst*XI (5′) and *Eco*RV (3′) restriction sites, the PCR product was *Bst*XI – *Eco*RV digested and then cloned between the same positions into the type 2 EBNA-2 pBluescript vector with the engineered *Eco*RV sequence. For construction of chimaera 7, an artificial *Bst*BI site was inserted in the type 2 EBNA-2 pBluescript plasmid, at the end of the TAD sequence (codons 429-430). The RG, CR7 and TAD sequences were amplified by *Pfu*-PCR from the type 1 EBNA-2 vector, using primers with overhanging *Bst*XI (5′) and *Bst*BI (3′) sites. The PCR product was then cloned into the type 2 EBNA-2 plasmid, between the same restriction sites. The identity of all the chimaeric EBNA-2 sequences was screened by restriction mapping and by sequencing (data not shown). These were subcloned as *Eco*RI – *Not*I fragments from the pBluescript background into an intermediate vector (pSuper backbone) bearing the *Bgl*II – *Not*I region from the pAG1 vector (described above), which consists of one BamHI W repeat and the *Eco*RI – *Not*I EBNA-2 coding sequence. The *Bgl*II – *Not*I sequences, comprising the BamHI W repeat and the chimaeric EBNA-2 ORFs, were then cloned into the OriP-p294 MCS expression vector, between *Bam*HI and *Not*I sites. Transcription of the chimaeric EBNA-2 ORFs in these plasmids is driven by the EBV Wp promoter, similarly to the type 1 and type 2 EBNA-2 constructs.

The pSNOC vector expressing a type 1 3-repeat EBNA-LP coding sequence is described elsewhere [116]. Briefly, this OriP expression plasmid contains the G418-resistance locus and the CMV immediate early promoter and the SV-40 poly A sequences, expressing the EBNA-LP coding sequence. To generate a homologous type 2 3-repeat EBNA-LP-expressing plasmid, a cDNA clone from the type 2 cell line C2+BL16 was obtained by *Pfu*-PCR using oligonucleotides 5′- ATG CGG CCA TGT AGG CCC ACT T -3′ and 5′- TGC CCA ACC ACA GGT TCA GGC A-3′, complementary to sequences within the C1 and YH exons, respectively, of AG876 EBV strain (coordinates 11403-11425 and 36141-36119). The amplified product was subcloned into pCR2.1-TOPO vector (Invitrogen) via TA cloning strategy and sequenced to verify its identity to the published AG876 EBNA-LP sequence. The type 2 3-repeat EBNA-LP cDNA was then cloned as an *Eco*RI fragment into the pSNOC empty vector.

The p554-4 plasmid, bearing the coding sequences for a type 1 ER-EBNA-2 and 2 BamHI W repeats was a kind gift from Bettina Kempkes [86], [115]. The p554-4 derivative expressing type 2 ER-EBNA-2 is described elsewhere [26]. These two vectors were used to generate the stable cell lines P3HR1 cl.16:ER-EBNA-2 T1/T2 (described below).

### Construction and characterisation of EBNA-2 EBV-BAC mutant

EBNA-2 in the type 1 B95-8 EBV-BAC [117] was substituted with the type 2 EBNA-2 gene by RecA-mediated homologous recombination. The final targeting construct used for this was cloned between the *Bam*HI and *Psp*0MI sites of pKov-Kan-ΔCm p4487.1 [118] and was called p121 (Figure S6). Starting from the type 2 EBNA-2 pBluescript plasmid (described above), type 1 upstream flanking sequences were added by cloning a *Hind*III – *Eco*RI fragment from the p554 vector [86], [115] using a *Hind*III site in the Bluescript polylinker. Type 1 downstream flanking sequence was then added by substituting in a *Pme*I to *Not*I fragment from p554. An *Xho*I site in the pBluescript polylinker upstream of the *Hind*III site was converted into a *Bgl*II site using an oligonucleotide adaptor and then the whole assembly was cloned as a *Bgl*II – *Not*I fragment between the *Bam*HI and *Psp*0MI sites of p4487.1. The resulting type 2 EBNA-2 with type 1 flanking sequences contained type 2 EBV from 36201 – 38008 of the AG876 sequence [119]. B95-8 EBV-BAC (p2089, chloramphenicol resistant) in *E. Coli* strain DH10B was kindly provided by W. Hammerschmidt [117]. For clarity, the name T1 E2 EBV-BAC is used for this plasmid in this paper. RecA recombination to produce the type 2 EBNA-2 BAC mutant used the same methods as described previously for construction of EBER mutants [120].

The integrity of the type 2 BAC was screened at each stage of recombination by miniprep DNA isolation, restriction digest and pulsed field gel electrophoresis (PFGE) in order to control for any second-site mutations that may have occurred. Substitution of the type 1 EBNA-2 ORF with the type 2 sequence introduced an additional *Eco*RI restriction site into the EBV-BAC, in front of the starting ATG of EBNA-2 open reading frame. Digestion with this restriction enzyme followed by PFGE analysis therefore enabled diagnosis of correct insertion of the type 2 EBNA-2 sequence (data not shown). *Eco*RI digestion revealed also that the type 2 EBNA-2 construct had lost 2 copies (out of 6) of the BamHI W repeat compared to the parental type 1 construct. This was confirmed by digestion with another restriction enzyme, *Eco*RV (data not shown) and was the only gross rearrangement that had occurred, as the pattern of the remaining bands was identical in the type 1 and the type 2 EBNA-2 BAC in both *Eco*RI and *Eco*RV digestions (data not shown). Because the number of BamHI W repeats varies considerably in different EBV strains, from 3 to 12 [94] and because the repeat number found in the type 2 BAC is within this range, this deletion was not considered to be undesirable.

### Establishment of 293 stable cell lines containing EBV-BAC with type 2 EBNA-2

EBV-BAC DNA was purified using EndoFree plasmid Maxi kit (Qiagen) and 1 µg was transfected into 293 cells with Lipofectamine2000 (Invitrogen) according to the manufacturer's instructions. Stable 293 cell clones were isolated under Hygromycin B selection (100 µg/ml), clonal cell lines were expanded and these were then screened for integrity of the EBV-BAC DNA by episome rescue and restriction digestion or PCR analysis (data not shown). A 293 clone containing the T1 E2 EBV-BAC, used to generate the type 2 BAC, was also produced.

### Virus production and titration

Infectious virus was produced from the 293 stable cell lines containing either T1 E2 EBV-BAC (with type 1 EBNA-2) or T2 E2 EBV-BAC (with type 2 EBNA-2) by transient transfection of BZLF1 and BALF4-expressing plasmids using Lipofectamine2000 (Invitrogen) [117], [121], [122]. After 4 days, the virus-containing supernatants were harvested, filtered (0.45 µm pore size) and titred by infecting Raji cells and counting GFP-positive cells (Green Raji Units, GRU) on an inverted fluorescent microscope, following treatment with 5 nM TPA (Sigma) and 5 mM sodium butyrate (Sigma), to enhance GFP expression.

### Purification of B cells and virus infections

Primary B cells were isolated from mixed-donors buffy coat residues by negative selection using RosetteSEP Human B cell Enrichment cocktail (Stemcell Technologies) as described by the manufacturer's instructions. The isolated cells were analyzed by flow cytometry for purity using fluorescein-isothiocyanate-conjugated anti-CD20 antibody (Dako). 2×10^6^ cells were infected with 5000 GRUs of recombinant viruses and cultured in RPMI 1640 medium supplemented with 20% FBS and antibiotics. At the desired time-points cells were harvested and processed for RNA or protein extraction. For LCL establishment, 10^6^ cells were infected with 5-fold serial dilutions of recombinant EBVs starting with 8000 GRUs. Cells were grown initially in RPMI 1640 medium with 20% FBS and antibiotics and every 2-3 days half the medium was replaced. Once LCLs had grown out in large culture volumes, the FBS level in the medium was reduced to 10% and cells were selected in Hygromycin B (Roche) at a concentration of 150 µg/ml for 3 weeks. After this time, hygromycin selection was discontinued and cells were not used for experimental investigation during hygromycin selection.

### Transfections

To generate P3HR1 cl.16:ER-EBNA-2 T1/T2 stable cell lines, 2×10^6^ cells were transfected with 4 µg of p554-4 [86], [115] or pERT2 [26] plasmids expressing ER-EBNA-2 type 1 and type 2 respectively using Neon transfection system (Invitrogen) set at 1300 V, 30 pulse width, 1 pulse. 24 hours after transfection G418 (Calbiochem) selection was added at a concentration of 500 µg/ml. Selected cell lines were grown out and tested for expression of ER-EBNA-2 T1/T2 by western blotting.

For EBNA-2/EBNA-LP cooperation assays in Daudi cells, the Neon system (Invitrogen) was used to transiently transfect 2×10^6^ cells with 4 µg of the OriP-p294 plasmids expressing type 1 or type 2 EBNA-2 and 2.4 µg DNA of the pSNOC plasmids expressing either type 1 or type 2 EBNA-LP. Empty vector (OriP-p294 or pSNOC) was added to normalize the total amount of DNA transfected (6.2 µg). Electroporation conditions were 1400 V, 30 pulse width and 1 pulse, which routinely gave approximately 40% transfection efficiency (assessed as GFP-positive cells when transfecting a GFP-expressing plasmid) and 80% of cell viability.

For the EREB2.5 growth assay, 6×10^6^ EREB2.5 cells were transfected with 5 µg of OriP-p294 plasmids bearing type 1/type 2 EBNA-2 sequences, either wild-type or chimaeric, using the Amaxa system (Lonza), program A-23. β-oestradiol was withdrawn immediately after transfection in order to select for β-oestradiol – independent lymphoblastoid cell lines. Cells were incubated overnight in 2 ml of complete medium supplemented with 20% FBS and antibiotics, but without β-oestradiol, in 12-well plates. The following day each transfected sample was diluted up to 10 ml with culture medium and plated into 5 wells of a 24-well plate. At the desired time-points cell samples were harvested for cell count analysis and RNA or protein extraction.

For immunofluorescence experiments, 5×10^6^ HeLa cells, grown on coverslips (VWR) in 6-well dishes, were transfected with 4 µg of OriP-p294 plasmids encoding either type 1, type 2 EBNA-2 or the chimaeric EBNA-2 proteins using Lipofectamine2000 (Invitrogen) as recommended by the manufacturer's instructions.

### RNA extraction and qRT-PCR

Total cell RNA was extracted from growing cells using Trizol reagent (Invitrogen) and then quantified by measuring its absorbance at 260 nm. RNA samples were digested with RQ1 RNase-free DNase (Promega). cDNA was prepared using Protoscript M-Mulv First strand cDNA Synthesis kit (New England Biolabs) using random primer as recommended by the manufacturer's instructions. To detect the expression of the EBNA-2-regulated genes, quantitative RT-PCR (qRT-PCR) was used with the primer pairs listed in Table S2. qRT-PCR was performed on an ABI 7900HT real-time PCR machine using Absolute QPCR SYBR Green ROX Mix (Thermo Scientific). The cycling conditions were: 95°C for 15 min, followed by 40 cycles of 15 sec at 95°C, 30 sec at 60°C and 40 sec at 72°C on a fast block. Dissociation curve analysis was performed at the end of each run to ensure absence of non-specific products. Quantification of mRNA levels was carried out using the Delta-Delta Ct method. Results were analyzed with SDS2.3 software (Applied Biosystems). mRNA levels for each target gene analyzed were normalized on the housekeeping gene GAPDH used as a loading control.

### RNase protection assay (RPA)

The LMP-1-specific probe for use in RPA was generated by PCR of genomic DNA derived from Daudi cells using the following primers, designed based on the EBV genome sequence: forward 5′-CAC TCA TAA CGA TGT ACA GC-3′ (coordinates 168881 to 169000 in EBV wild-type, accession number NC007605) and reverse 5′- CAA GAA ACA CGC GTT ACT C-3′ (coordinates 169103 to 169121 in EBV wild-type, accession number NC007605). The PCR product (241 bp) was cloned into pCR2.1-TOPO vector and sequenced and the plasmid was then linearized by BamHI digestion at the 5′ end of the PCR product. 1 µg of the linearized plasmid was *in vitro*-transcribed with T7 enzyme using the MAXIscript SP6/T7 *in vitro* transcription kit (Ambion), according to the manufacturer's instructions, for production of ^32^P-labelled antisense RNA probe. RPAs were carried out using RPAIII kit (Ambion) as recommended by the manufacturer's instructions. Briefly, 20 µg of total cell RNA were hybridized overnight at 42°C with 50000 cpm of the probe. As negative control, an equivalent amount of yeast RNA was included in a hybridization reaction. Single-stranded RNA was digested with an RNase A/T1 mixture for 30 min at 37°C. Protected fragments were precipitated and separated on a polyacrylamide gel and the gel was analyzed on a phosphoimager.

### Immunoblotting and antibodies

Cell samples were lysed for 15 min on ice in 2 volumes of RIPA lysis buffer (150 mM NaCl, 1% v/v Nonidet-P40, 0.5% v/v deoxycholic acid, 0.1% w/v SDS, 50 mM Tris/HCl, pH 8.0) supplemented with 1 mM PMSF (Fluka) and Complete protease inhibitors (Roche). Samples were centrifuged at 16000 g for 15 min, to pellet cell debris, and the supernatant was harvested. Proteins were fractionated by SDS-PAGE and transferred onto a nitrocellulose membrane. After blocking for 1 h at room-temperature with 5% milk powder in PBS/0.1% Tween-20 (Sigma), membranes were incubated overnight at 4°C with the primary antibody diluted in blocking buffer. The primary antibodies used are listed in Table S1. The secondary antibodies were horseradish peroxidise-conjugated anti-mouse (GE Healthcare) or anti-rabbit or anti-human IgG (Dako) and were used at a dilution of 1/2000 for 1 h at room-temperature. Bound immunocomplexes were detected by using ECL western blotting detection reagents (Amersham).

### Indirect immunofluorescence

24 hours after transfection, HeLa cells were fixed using 4% paraformaldehyde and permeabilized with 0.25% Triton-X 100 (Sigma) in PBS. Primary anti-EBNA-2 antibody (PE2 clone) was diluted 1/100 in blocking buffer (1% bovine serum albumin in PBS) and cells were incubated for 45 min at room-temperature. The coverslips were washed 3 times with PBS and TRITC-conjugated anti-mouse secondary antibody (Sigma) was applied at a dilution of 1/5000 in blocking buffer for 45 min at room-temperature. After 3 washes in PBS the coverslips were mounted in Mowiol and visualized using a Zeiss 510 Meta laser confocal microscope.

### Accession numbers

Database entries for genes mentioned in this manuscript include: CXCR7 (RefSeq NM 020311.2), EBV type 1 (accession number AJ507799) and EBV type 2 (accession number DQ279927). Annotation in the EBV genome shows EBNA-2 and LMP-1 genes.

## Supporting Information

Figure S1 **Analysis of EBNA-LP species expressed in Daudi cells and Daudi:ER-EBNA-2 T1/T2 stable cell lines.** Protein extracts were prepared from Daudi:ER-EBNA-2 T1/T2 stable cell lines treated with oestrogen (est) for 4 hours (+) or left untreated (-) and from Daudi cells. Western blot analysis was performed using anti-EBNA-2 (PE2) and anti-EBNA-LP (JF186 and 4D3) antibodies. In Daudi cells no EBNA-2 was detected, because of the deletion that encompasses the EBNA-2 locus and the Y1Y2 exons of EBNA-LP. No EBNA-LP was detected with the type 1-specific JF186 antibody, confirming that EBNA-LP in these cells is type 2. The 4D3 antibody recognized the 37 kDa EBNA-LP species, which corresponds to a 4-repeat isoform and lacks Y1 and Y2 domains (marked by the arrow). In the stable cell lines bearing the ER-tagged EBNA-2 proteins, treatment with oestrogen produced a clear increase in abundance of the fusion proteins. This was also accompanied by a shift in the electrophoretic mobility, which is due to phosphorylation [115]. Full-length type 1 EBNA-LP species with 2, 3 and 4 repeats (2R, 3R and 4R) were detected with both JF186 and 4D3 antibodies, indicating that they are expressed from the p554-4 plasmid. Lysates from B95-8 cells and 293 expressing 3 or 7-repeat EBNA-LP type 1 (T1 ELP 3R and 7R) were used as size markers to determine the number of repeats in EBNA-LP proteins. β-actin immunoblotting was performed to ensure equal loading of the proteins. Numbers on the left hand-side of the EBNA-LP immunoblots represent molecular weight (in kDa).
(TIF)

Figure S2 **Analysis of EBNA-LP species expressed in P3HR1 cl.16 cells and P3HR1 cl.16:ER-EBNA-2 T1/T2 stable cell lines.** P3HR1 cl.16:ER-EBNA-2 T1/T2 stable cell lines were treated with oestrogen (est) for 4 hours (+) or left untreated (-) and proteins were extracted and analyzed by western blotting. Protein samples from untreated P3HR1 cl.16 cells were also included in the analysis. EBNA-2 was detected with the PE2 antibody and EBNA-LP with the JF186 and 4D3 antibodies. Similarly to Daudi cells, no EBNA-2 was detected in P3HR1 cl.16 cells, because of the deletion, and no EBNA-LP was detected with type 1-specific JF186 antibody, confirming that EBNA-LP in these cells is type 2. A major ∼30 kDa EBNA-LP species and a minor ∼50 kDa isoform were detected with the 4D3 antibody: they both lack the carboxyl-terminal Y1Y2 region and they comprise 3 and 6 repeats (marked by arrows). In the stable cell lines bearing the ER-conjugated EBNA-2 proteins, both JF186 and 4D3 antibodies detected full-length type 1 EBNA-LP species with 2, 3, 4, 5, 6 and 7 repeats (2R, 3R, 4R, 5R, 6R and 7R) expressed from the p554-4 mini-EBV genome. Protein samples from B95-8 cells and 293 expressing 3 or 7-repeat EBNA-LP type 1 (T1 ELP 3R and 7R) were used as size markers for EBNA-LP repeats number. β-actin immunoblotting was used as protein loading control. Numbers on the left hand-side of the EBNA-LP blots represent protein molecular weight (in kDa).
(TIF)

Figure S3 **Live cell counts of type 1 and type 2 EBV-BAC transformants.** 10^6^ primary B cells were either left uninfected (mock) or infected with 5-fold serial dilutions of recombinant EBVs starting with 8000 GRUs of T1 or T2 E2 EBV-BAC recombinant viruses, which express either type 1 or type 2 EBNA-2 respectively. Infected cells were maintained in culture over time in order to establish LCLs and 1 month after infection differences in cell proliferation levels were assessed by counting the number of live cells on a haemocytometer. Error bars represent standard deviations. Data from 1 representative experiment of 2 is shown.
(TIF)

Figure S4 **Validation of T1 and T2 E2 EBV-BAC LCLs.** (**A**) Western blot analysis of latency-associated EBV proteins in LCLs established with type 1 (T1) and type 2 (T2) EBNA-2 (E2) EBV-BAC viruses. There were no major differences in EBV latent proteins expression levels between type 1 and type 2 LCLs for most of the antigens examined. An exception is represented by EBNA-LP, as a 3-repeat isoform was detected in the type 2 LCL, whereas type 1 transformants expressed EBNA-LP species with 5 and 4 repeats and in one of the type 1 clone also a 3-repeat isoform was detected. In both viruses the EBNA-LP is type 1 (detected by JF186 antibody). Western blot analysis of an independent type 2 LCLs confirmed expression of an EBNA-LP protein with 3 repeats (data not shown). This is consistent with the loss of 2 BamHI W repeats, relative to the parental type 1 EBV BAC construct (described in Materials and Methods). This variation is not likely to affect transformation efficiency of the two types of recombinant viruses, since EBNA-LP proteins with a number of repeats above 2 have been shown to be functionally equivalent at enhancing EBNA-2-mediated activation of LMP-1 [17], [60]. (**B**) Western blot analysis of EBNA-3A and -3C in additional type 1 (T1) and type 2 (T2) LCLs. Minor variations in expression levels were detected in EBNA-3A and -3C across all the clones examined **(A) and (B)**, but these do not seem to be consistently linked to one specific type of LCL. Re-probing with anti-β-actin antibody ensured that equal amounts of proteins were loaded on the gel.
(TIF)

Figure S5 **Nuclear localization of the chimaeric EBNA-2 proteins (A) and wild-type type 1 and type 2 (B) in HeLa cells.** OriP-p294 plasmids either bearing chimaeric EBNA-2 sequences (chimaera 1 to 7, panel A) or expressing type 1 (E2T1) or type 2 (E2T2) EBNA-2 (panel B) were transiently transfected into HeLa cells. After 24 hours, cells were fixed, permeabilized and probed with the anti-EBNA-2 antibody (PE2 clone) followed by TRITC-conjugated anti-mouse secondary antibody. The location of the EBNA-2 proteins was assessed by confocal microscopy. All the chimaeric EBNA-2 proteins were localized to the nucleus, with exclusion from nucleoli, as seen for wild-type type 1 and type 2. e.v.: empty vector-transfected cells; n.t.: non-transfected cells; E2T1 + II Ab: cells transfected with type 1 EBNA-2-expressing plasmid and stained with the secondary antibody only.
(TIF)

Figure S6 **Schematic of the EBV sequences used to generate the type 2 EBNA-2 BAC mutant.** The boxed region shows the part of the EBV genome cloned into the targeting vector p121 with the EBV type sequence indicated below. The filled box marks the EBNA-2 coding region.
(TIF)

Table S1 **Primary antibodies for immunoblotting.**
(DOCX)

Table S2 **qRT-PCR primer sequences to detect EBNA2-regulated genes.**
(DOCX)
