# Supplementary figures and images for: C-Terminal Region of EBNA-2 Determines the Superior Transforming Ability of Type 1 Epstein-Barr Virus by Enhanced Gene Regulation of LMP-1 and CXCR7
Source: PLoS Pathog. 2011 Jul 28;7(7):e1002164. doi: 10.1371/journal.ppat.1002164 (PMC3145799; doi:10.1371/journal.ppat.1002164)

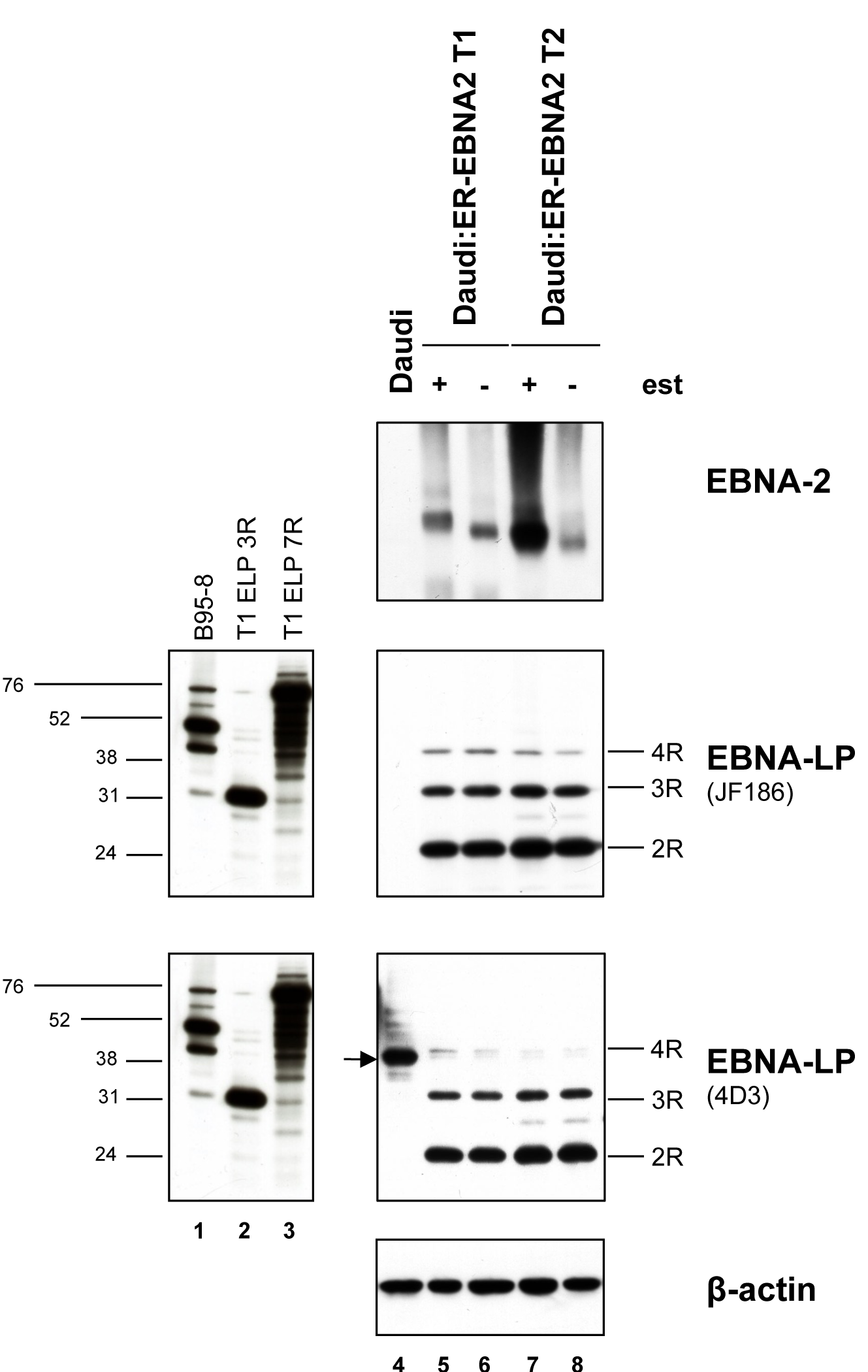

Supplement: Figure S1 — Analysis of EBNA-LP species expressed in Daudi cells and Daudi:ER-EBNA-2 T1/T2 stable cell lines. Protein extracts were prepared from Daudi:ER-EBNA-2 T1/T2 stable cell lines treated with oestrogen (est) for 4 hours (+) or left untreated (-) and from Daudi cells. Western blot analysis was performed using anti-EBNA-2 (PE2) and anti-EBNA-LP (JF186 and 4D3) antibodies. In Daudi cells no EBNA-2 was detected, because of the deletion that encompasses the EBNA-2 locus and the Y1Y2 exons of EBNA-LP. No EBNA-LP was detected with the type 1-specific JF186 antibody, confirming that EBNA-LP in these cells is type 2. The 4D3 antibody recognized the 37 kDa EBNA-LP species, which corresponds to a 4-repeat isoform and lacks Y1 and Y2 domains (marked by the arrow). In the stable cell lines bearing the ER-tagged EBNA-2 proteins, treatment with oestrogen produced a clear increase in abundance of the fusion proteins. This was also accompanied by a shift in the electrophoretic mobility, which is due to phosphorylation [115]. Full-length type 1 EBNA-LP species with 2, 3 and 4 repeats (2R, 3R and 4R) were detected with both JF186 and 4D3 antibodies, indicating that they are expressed from the p554-4 plasmid. Lysates from B95-8 cells and 293 expressing 3 or 7-repeat EBNA-LP type 1 (T1 ELP 3R and 7R) were used as size markers to determine the number of repeats in EBNA-LP proteins. β-actin immunoblotting was performed to ensure equal loading of the proteins. Numbers on the left hand-side of the EBNA-LP immunoblots represent molecular weight (in kDa). (TIF) [file ppat.1002164.s001.tif]

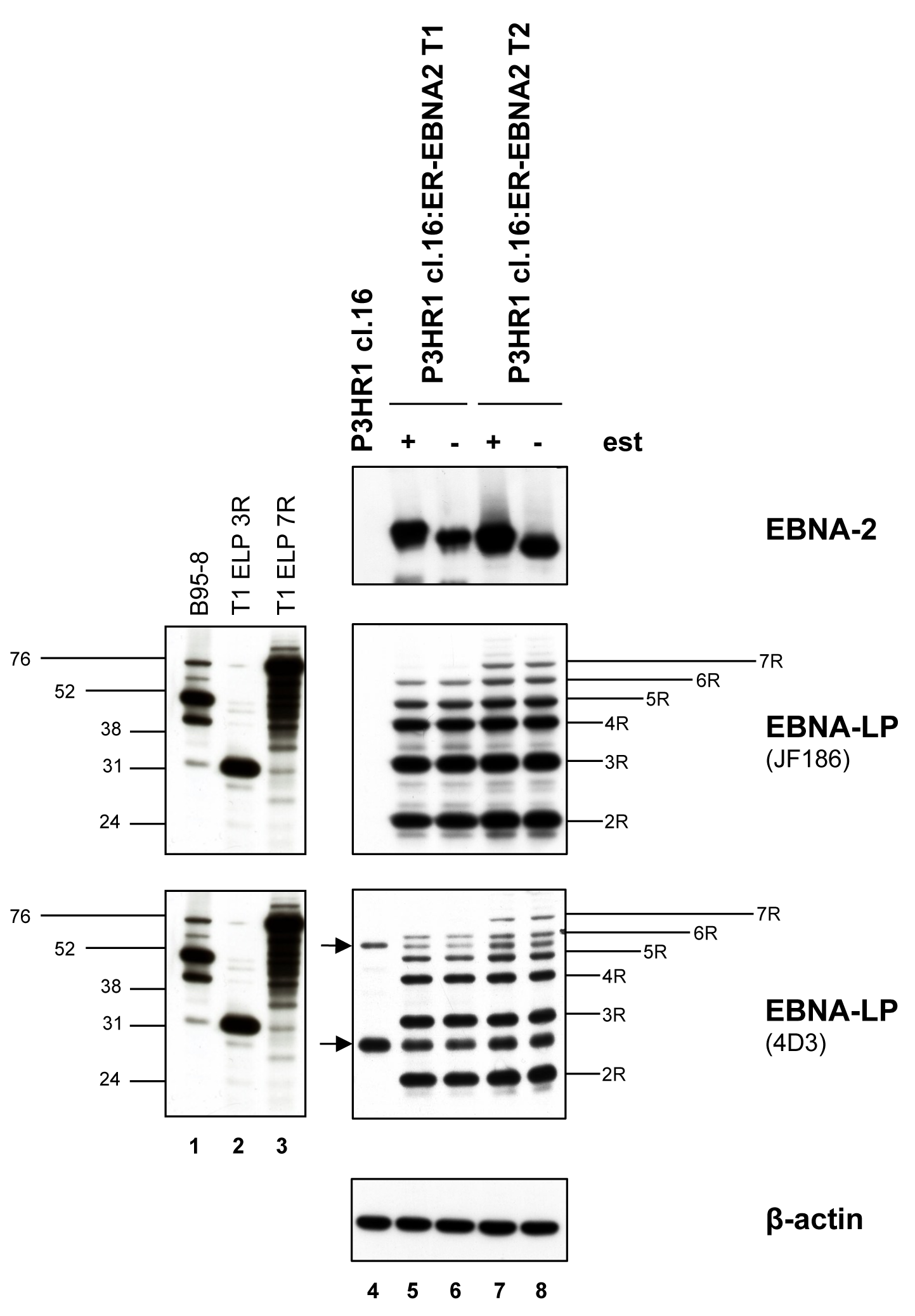

Supplement: Figure S2 — Analysis of EBNA-LP species expressed in P3HR1 cl.16 cells and P3HR1 cl.16:ER-EBNA-2 T1/T2 stable cell lines. P3HR1 cl.16:ER-EBNA-2 T1/T2 stable cell lines were treated with oestrogen (est) for 4 hours (+) or left untreated (-) and proteins were extracted and analyzed by western blotting. Protein samples from untreated P3HR1 cl.16 cells were also included in the analysis. EBNA-2 was detected with the PE2 antibody and EBNA-LP with the JF186 and 4D3 antibodies. Similarly to Daudi cells, no EBNA-2 was detected in P3HR1 cl.16 cells, because of the deletion, and no EBNA-LP was detected with type 1-specific JF186 antibody, confirming that EBNA-LP in these cells is type 2. A major ∼30 kDa EBNA-LP species and a minor ∼50 kDa isoform were detected with the 4D3 antibody: they both lack the carboxyl-terminal Y1Y2 region and they comprise 3 and 6 repeats (marked by arrows). In the stable cell lines bearing the ER-conjugated EBNA-2 proteins, both JF186 and 4D3 antibodies detected full-length type 1 EBNA-LP species with 2, 3, 4, 5, 6 and 7 repeats (2R, 3R, 4R, 5R, 6R and 7R) expressed from the p554-4 mini-EBV genome. Protein samples from B95-8 cells and 293 expressing 3 or 7-repeat EBNA-LP type 1 (T1 ELP 3R and 7R) were used as size markers for EBNA-LP repeats number. β-actin immunoblotting was used as protein loading control. Numbers on the left hand-side of the EBNA-LP blots represent protein molecular weight (in kDa). (TIF) [file ppat.1002164.s002.tif]

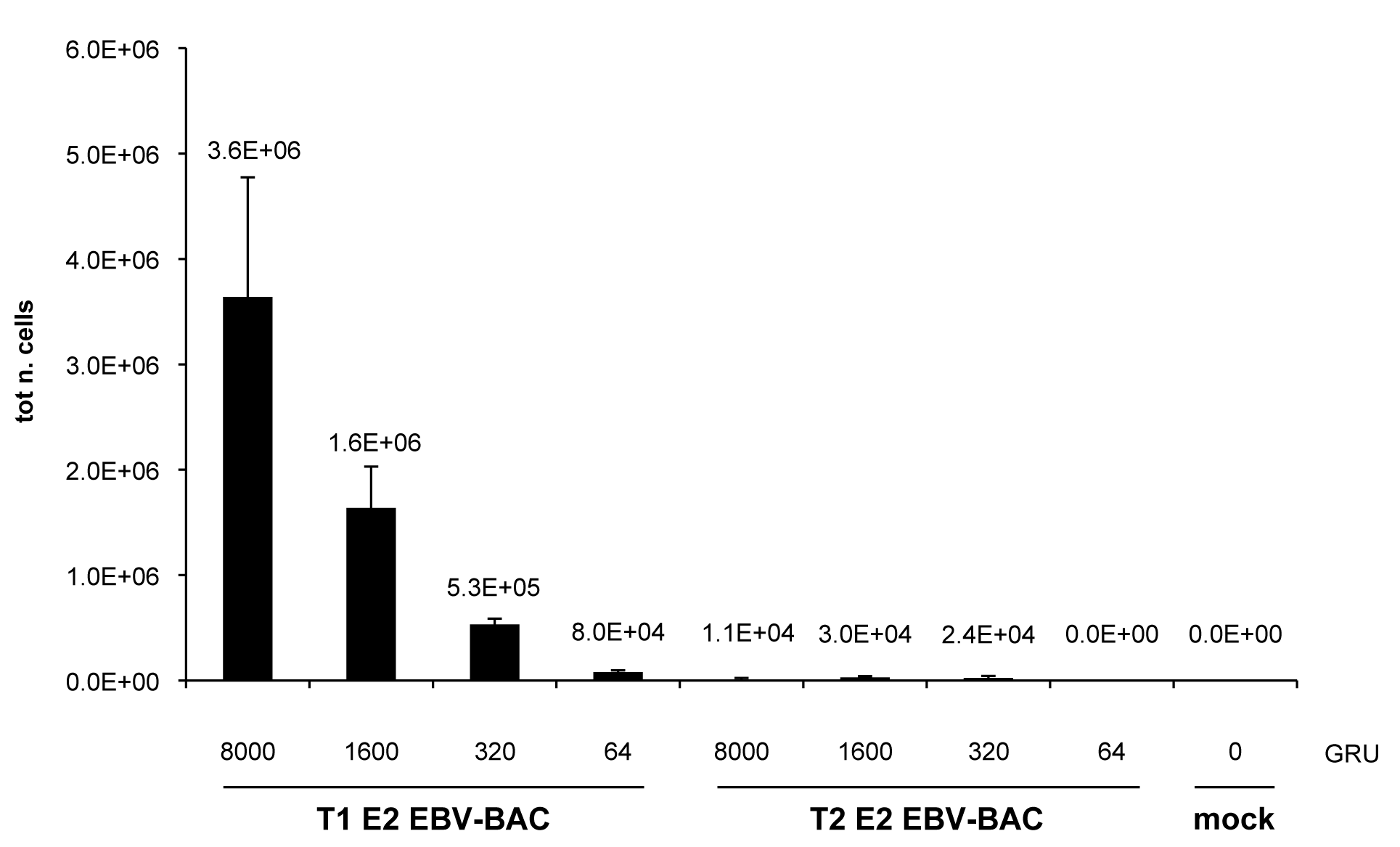

Supplement: Figure S3 — Live cell counts of type 1 and type 2 EBV-BAC transformants. 106 primary B cells were either left uninfected (mock) or infected with 5-fold serial dilutions of recombinant EBVs starting with 8000 GRUs of T1 or T2 E2 EBV-BAC recombinant viruses, which express either type 1 or type 2 EBNA-2 respectively. Infected cells were maintained in culture over time in order to establish LCLs and 1 month after infection differences in cell proliferation levels were assessed by counting the number of live cells on a haemocytometer. Error bars represent standard deviations. Data from 1 representative experiment of 2 is shown. (TIF) [file ppat.1002164.s003.tif]

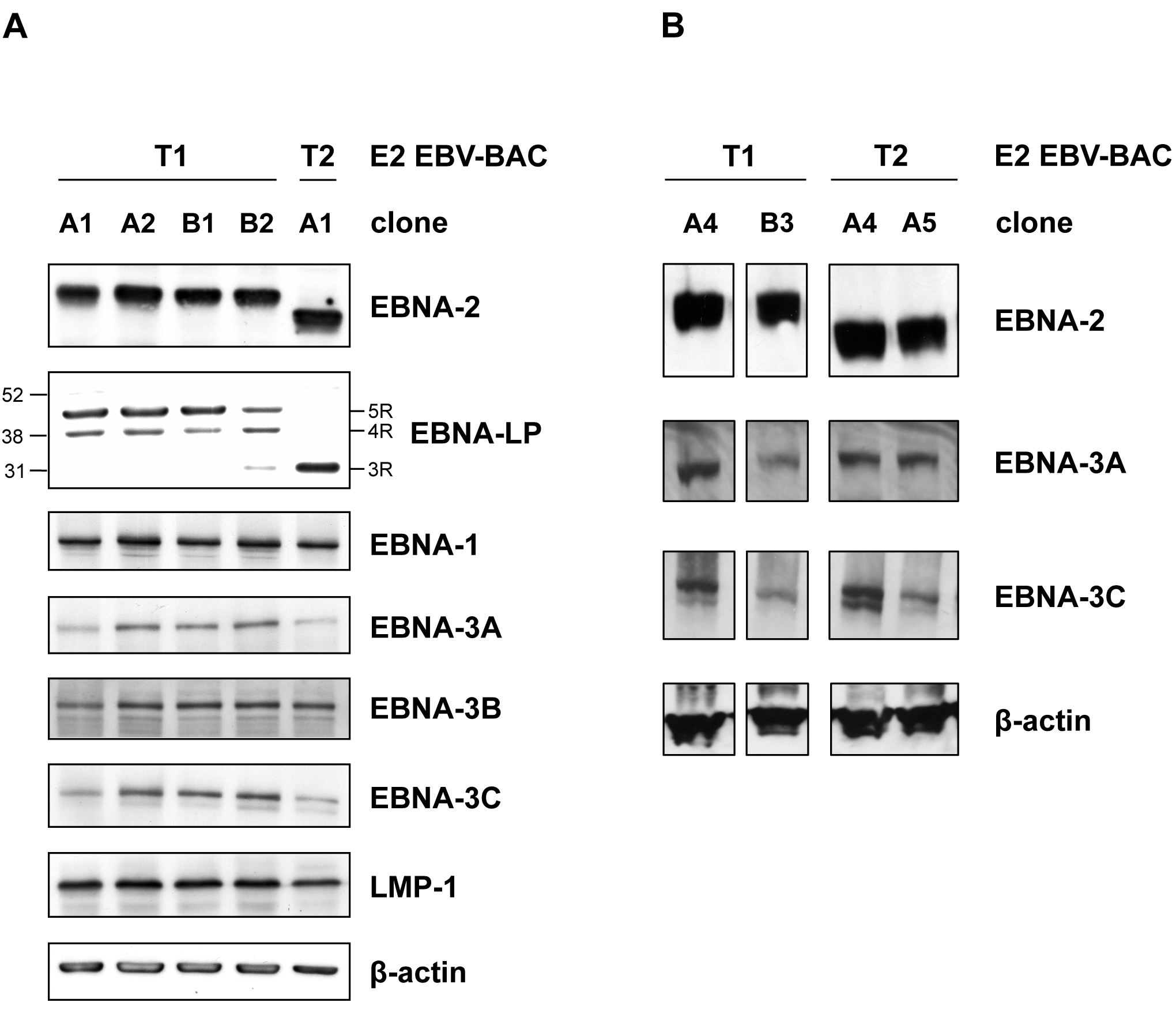

Supplement: Figure S4 — Validation of T1 and T2 E2 EBV-BAC LCLs. (A) Western blot analysis of latency-associated EBV proteins in LCLs established with type 1 (T1) and type 2 (T2) EBNA-2 (E2) EBV-BAC viruses. There were no major differences in EBV latent proteins expression levels between type 1 and type 2 LCLs for most of the antigens examined. An exception is represented by EBNA-LP, as a 3-repeat isoform was detected in the type 2 LCL, whereas type 1 transformants expressed EBNA-LP species with 5 and 4 repeats and in one of the type 1 clone also a 3-repeat isoform was detected. In both viruses the EBNA-LP is type 1 (detected by JF186 antibody). Western blot analysis of an independent type 2 LCLs confirmed expression of an EBNA-LP protein with 3 repeats (data not shown). This is consistent with the loss of 2 BamHI W repeats, relative to the parental type 1 EBV BAC construct (described in Materials and Methods). This variation is not likely to affect transformation efficiency of the two types of recombinant viruses, since EBNA-LP proteins with a number of repeats above 2 have been shown to be functionally equivalent at enhancing EBNA-2-mediated activation of LMP-1 [17], [60]. (B) Western blot analysis of EBNA-3A and -3C in additional type 1 (T1) and type 2 (T2) LCLs. Minor variations in expression levels were detected in EBNA-3A and -3C across all the clones examined (A) and (B), but these do not seem to be consistently linked to one specific type of LCL. Re-probing with anti-β-actin antibody ensured that equal amounts of proteins were loaded on the gel. (TIF) [file ppat.1002164.s004.tif]

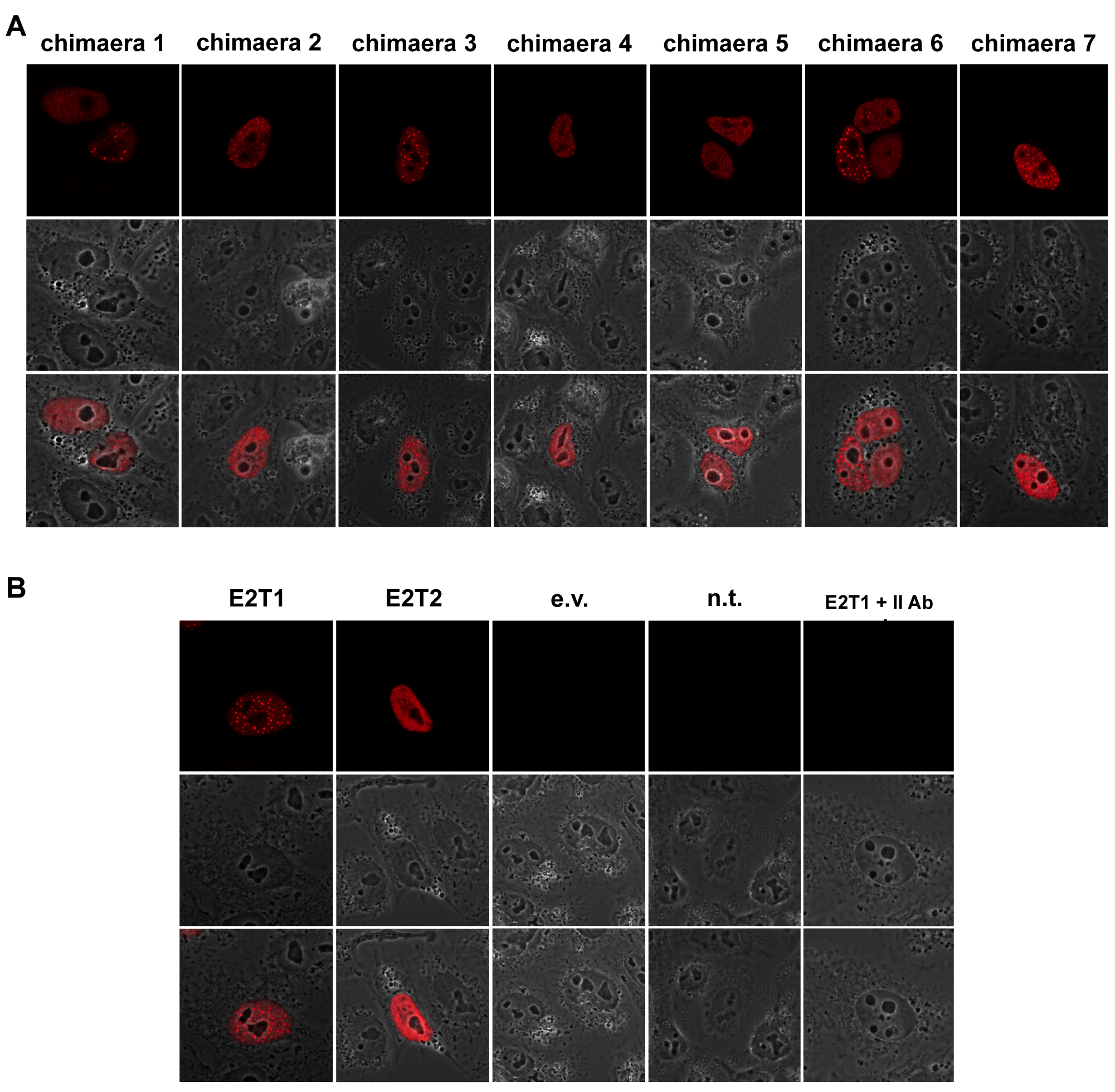

Supplement: Figure S5 — Nuclear localization of the chimaeric EBNA-2 proteins (A) and wild-type type 1 and type 2 (B) in HeLa cells. OriP-p294 plasmids either bearing chimaeric EBNA-2 sequences (chimaera 1 to 7, panel A) or expressing type 1 (E2T1) or type 2 (E2T2) EBNA-2 (panel B) were transiently transfected into HeLa cells. After 24 hours, cells were fixed, permeabilized and probed with the anti-EBNA-2 antibody (PE2 clone) followed by TRITC-conjugated anti-mouse secondary antibody. The location of the EBNA-2 proteins was assessed by confocal microscopy. All the chimaeric EBNA-2 proteins were localized to the nucleus, with exclusion from nucleoli, as seen for wild-type type 1 and type 2. e.v.: empty vector-transfected cells; n.t.: non-transfected cells; E2T1 + II Ab: cells transfected with type 1 EBNA-2-expressing plasmid and stained with the secondary antibody only. (TIF) [file ppat.1002164.s005.tif]

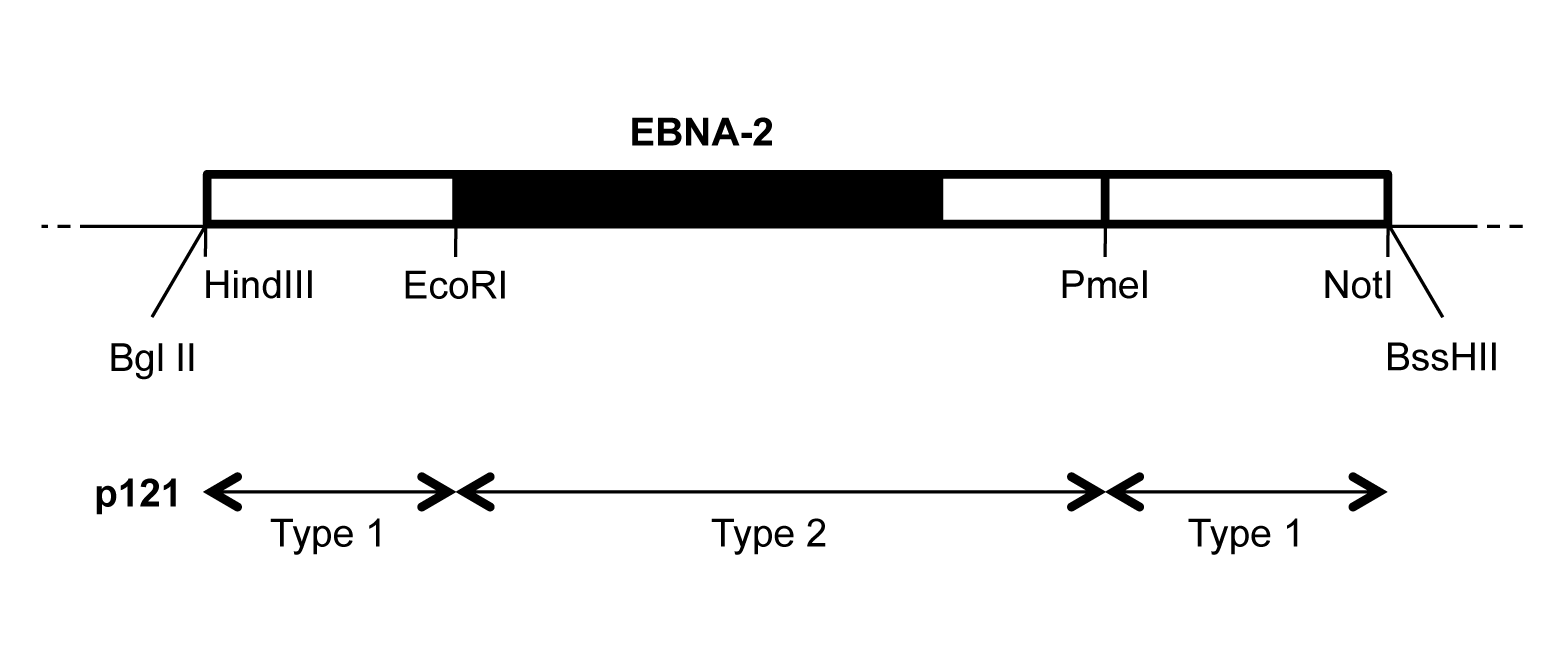

Supplement: Figure S6 — Schematic of the EBV sequences used to generate the type 2 EBNA-2 BAC mutant. The boxed region shows the part of the EBV genome cloned into the targeting vector p121 with the EBV type sequence indicated below. The filled box marks the EBNA-2 coding region. (TIF) [file ppat.1002164.s006.tif]
