# Supplementary material for: C-Terminal Region of EBNA-2 Determines the Superior Transforming Ability of Type 1 Epstein-Barr Virus by Enhanced Gene Regulation of LMP-1 and CXCR7
Source: PLoS Pathog. 2011 Jul 28;7(7):e1002164. doi: 10.1371/journal.ppat.1002164 (PMC3145799; doi:10.1371/journal.ppat.1002164)
Supplement: Table S1 — Primary antibodies for immunoblotting. (DOCX) [file ppat.1002164.s007.docx]

**Table S1. Primary antibodies for immunoblotting**

| **Antigen** | **Primary antibody** | **Source** | **Dilution** |
| --- | --- | --- | --- |
| **EBNA-2** | PE2, mouse monoclonal ^1^  (recognizes both types with equal efficiency on a western blot; Lucchesi *et al.*, 2008) | Tissue culture supernatant from hybridoma cell line |  |
| **EBNA-LP** | JF186, mouse monoclonal, (recognizes only type 1) | Tissue culture supernatant from hybridoma cell line (Finke *et al.*, 1987) |  |
|  | 4D3, mouse monoclonal, (recognizes both types) ^2^ | Shaku *et al.*, 2005 | 1/100000 |
| **EBNA-1** | human serum |  |  |
| **EBNA-3A** | sheep | ExAlpha, USA | 1/1000 |
| **EBNA-3B** | sheep | ExAlpha, USA | 1/350 |
| **EBNA-3C** | A-10, mouse monoclonal ^1^ | Tissue culture supernatant from hybridoma cell line |  |
| **LMP-1** | CS 1-4, mouse monoclonal | Dako | 1/500 |
| **β-actin** | AC-15, mouse monoclonal | Sigma | 1/2500 |
| **GAPDH** | Mouse monoclonal | Ambion | 1/500 |

^1^ kind gift from Prof. M. Rowe, University of Birmingham, UK

^2^ kind gift from Dr. Kawaguchi, University of Tokyo, Japan
