# Supplementary material for: C-Terminal Region of EBNA-2 Determines the Superior Transforming Ability of Type 1 Epstein-Barr Virus by Enhanced Gene Regulation of LMP-1 and CXCR7
Source: PLoS Pathog. 2011 Jul 28;7(7):e1002164. doi: 10.1371/journal.ppat.1002164 (PMC3145799; doi:10.1371/journal.ppat.1002164)
Supplement: Table S2 — qRT-PCR primer sequences to detect EBNA2-regulated genes. (DOCX) [file ppat.1002164.s008.docx]

**Table S2. qRT-PCR primer sequences to detect EBNA2-regulated genes**

| **Gene** | **Forward primer** | **Reverse primer** |
| --- | --- | --- |
| **LMP-1** | 5’-CTAGGAAGAAGGCTAGGAAG-3’ | 5’-CTAGGAAGAAGGCTAGGAAG-3’ |
| **CXCR7** | 5’-CAGCTTCAGATCTGGGTATTTATCC-3’ | 5’-TGGGCATGTTGGGACACATCACC-3’ |
| **CXCR4** | 5’-ATCTTCCTGCCCACCATCTACTCCATCATC-3’ | 5’-ATCCAGACGCCAACATAGACCACCTTTTCA-3’ |
| **GAPDH** | 5’-TGCCTCCTGCACCACCAACT-3’ | 5’-CGCCTGCTTCACCACCTTC-3’ |
